# Synergistic Performance Boosts of Dopamine‐Derived Carbon Shell Over Bi‐metallic Sulfide: A Promising Advancement for High‐Performance Lithium‐Ion Battery Anodes

**DOI:** 10.1002/advs.202308160

**Published:** 2024-02-11

**Authors:** Roshan Mangal Bhattarai, Nghia Le, Kisan Chhetri, Debendra Acharya, Sudhakaran Moopri Singer Pandiyarajan, Shirjana Saud, Sang Jae Kim, Young Sun Mok

**Affiliations:** ^1^ Department of Chemical Engineering Jeju National University 102 Jejudaehak‐ro Jeju 63243 Republic of Korea; ^2^ Department of Chemistry Mississippi State University PO Box 9573 Mississippi State MS 39762 USA; ^3^ Department of Nano Convergence Engineering Jeonbuk National University Jeonju 561756 Republic of Korea; ^4^ Regional Leading Research Center (RLRC) for Nanocarbon‐based Energy Materials and Application Technology Jeonbuk National University Jeollabuk‐do 54001 Republic of Korea; ^5^ School of Materials Science & Engineering Kookmin University Seoul 02707 Republic of Korea; ^6^ Nanomaterials and System Laboratory Department of Mechatronics Engineering Jeju National University 102 Jejudaehak‐ro Jeju 63243 Republic of Korea

**Keywords:** CoMoS, core‐shell, LIBs, N‐doped carbon, stability

## Abstract

A CoMoS composite is synthesized to combine the benefits of cobalt and molybdenum sulfides as an anodic material for advanced lithium‐ion batteries (LIBs). The synthesis is accomplished using a simple two‐step hydrothermal method and the resulting CoMoS nanocomposites are subsequently encapsulated in a carbonized polydopamine shell. The synthesis procedure exploited the self‐polymerization ability of dopamine to create nitrogen‐doped carbon‐coated cobalt molybdenum sulfide, denoted as CoMoS@NC. Notably, the de‐lithiation capacity of CoMoS and CoMoS@NC is 420 and 709 mAh g⁻^1^, respectively, even after 100 lithiation/de‐lithiation cycles at a current density of 200 mA g⁻^1^. Furthermore, excellent capacity retention ability is observed for CoMoS@NC as it withstood 600 consecutive lithiation/de‐lithiation cycles with 94% capacity retention. Moreover, a LIB full‐cell assembly incorporating the CoMoS@NC anode and an NMC‐532 cathode is subjected to comprehensive electrochemical and practical tests to evaluate the performance of the anode. In addition, the density functional theory showcases the increased lithium adsorption for CoMoS@NC, supporting the experimental findings. Hence, the use of dopamine as a nitrogen‐doped carbon shell enhanced the performance of the CoMoS nanocomposites in experimental and theoretical tests, positioning the material as a strong candidate for LIB anode.

## Graphical abstract

Graphene‐like nitrogen‐doped carbon shell encapsulated metal sulfide nanocomposite core for highly stable and superior capacity anode material for lithium‐ion battery application employing the intercalation and surface adsorption synergistic kinetics.

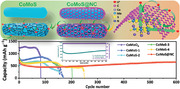

## 1 Introduction

Mitigation of the impact of carbon emissions to effectively combat global warming requires the adoption of environmentally friendly modes of transportation, such as electric vehicles, as an imperative step toward the reduction of global warming and climate change.^[^ ^1^ ^]^ Among various options, lithium‐ion batteries (LIBs) have emerged as highly desirable candidates for electric vehicles due to their high energy density, low self‐discharge, lightweight design, and numerous other advantages.^[^ ^2^ ^]^ Furthermore, LIBs have been widely used as a primary energy source for countless electronic devices for several decades.^[^ ^3^ ^]^ Given the extensive utilization of LIBs, it has become crucial for researchers and scientists to enhance the efficiency of these batteries.

Traditional LIBs employ graphite as the anodic material. However, graphite, with a theoretical specific capacity of 372 mAh g⁻^1^, can no longer meet the energy density requirements demanded by modern LIBs.^[^ ^4^ ^]^ Consequently, the past few decades have seen global efforts to explore alternative anodic materials capable of enhancing the capacity and cycling performance of LIBs.^[^ ^5^ ^]^ In this regard, considerable research has been devoted to developing transition metal sulfides as a viable substitute for graphite in anodic materials. This focus on transition metal sulfides is primarily attributable to their significant theoretical capacity, natural abundance, and minimal volumetric expansion during lithiation and de‐lithiation cycles.^[^ ^6^ ^]^ Particularly, layered metal chalcogenides, characterized by covalently bonded atoms that form stacked two‐dimensional layers via weak van der Waals interactions, have been identified to exhibit outstanding resilience against volume expansion.^[^ ^7^ ^]^ Consequently, they were shown to demonstrate enhanced cyclability as materials for LIBs.^[^ ^8^ ^]^ The layered structure of molybdenum sulfide endows it with a notable theoretical capacity of ≈669 mAh g⁻^1^, positioning it as a promising anodic material for LIBs.^[^ ^9^ ^]^ Nonetheless, its exceptional qualities are impeded by its subpar cyclability and low electrical conductivity.^[^ ^10^ ^]^ Conversely, cobalt sulfide, known for its comparatively high electronic conductivity and thermal stability, has been regarded as another useful material for LIB anodes.^[^ ^11^ ^]^ Nonetheless, akin to molybdenum sulfide, cobalt sulfide is also susceptible to pulverization resulting from volumetric fluctuations over numerous cycles.^[^ ^12^ ^]^

An alternative approach to augment the characteristics of cobalt and molybdenum sulfides involves their combination to create a di‐metal sulfide. This combination would not only increase the electronic conductivity of the composite but also introduce an additional redox reaction species to enhance the electrochemical performance of the energy storage device.^[^ ^13^ ^]^ Yet, the utilization of composites of cobalt, molybdenum, and sulfur in energy storage devices, such as a CoMoS composite for LIBs, has not received much attention. Lu et al. employed alginate‐derived biomass as a template to fabricate three‐dimensional (3D) MoS_2_@ADC and CoMoS_3.13_@ADC nanostructures.^[^ ^14^ ^]^ They found CoMoS_3.13_@ADC to have superior cycling stability and rate capability compared to MoS_2_@ADC during ≈150 cycles with different rate capabilities owing mainly to the addition of cobalt as a dopant and the 3D conductive alginate‐derived carbon matrix. Likewise, Liao et al. synthesized the Co_3_S_4_/CoMo_2_S_4_@rGO composite and reported a reversible capacity of 595.4 mAh g⁻^1^ at a current density of 0.2 A g⁻^1^ for 100 cycles.^[^ ^15^ ^]^

On the other hand, the practice of incorporating carbon into active composite materials has long been established and demonstrated to enhance the performance of energy storage devices in terms of stability and capacity. Dopamine, known for its nitrogen‐doped carbon‐rich properties, has been extensively investigated as a precursor in numerous applications.^[^ ^16^ ^]^ The porous morphology and multilayer graphene‐like structure of dopamine result in an electrical conductivity comparable to that of nitrogen‐doped graphene.^[^ ^17^ ^]^ Veerasubramani et al., in two separate works, reported their extensive exploration of the effect of using polydopamine as a coating for metal chalcogenide composites namely Cu_2_SnSe_4_ and SnS quantum flakes.^[^ ^18^ ^]^ In both of their studies, they found the capacity and stability of the N‐doped carbon‐coated metal chalcogenide composite to have improved owing to the ability of the N‐doped carbon coating to accommodate volumetric strain and enhance the ionic and electronic conductivity.

Hence, our pursuit of combining cobalt and molybdenum sulfides to exploit the best of their respective features led us to adopt a simple two‐step hydrothermal method to synthesize the CoMoS composite. The self‐polymerization ability of dopamine was employed to synthesize nitrogen‐doped carbon‐coated cobalt molybdenum sulfide, denoted as CoMoS@NC. The polydopamine shell encapsulating the CoMoS nanocomposites was subsequently carbonized to function as a buffering and conducting matrix. This matrix served to restrict the volumetric change and increase the electronic conductivity to elevate the specific capacity and long‐term stability of the CoMoS@NC as an anodic material for LIBs.

## 2 Results

### 2.1 Physical Characterization

**Figure 1:**
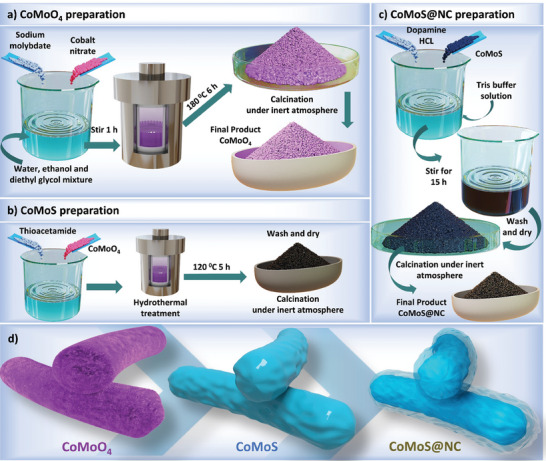
Schematic representation of different sample preparation techniques.

**Figure 2:**
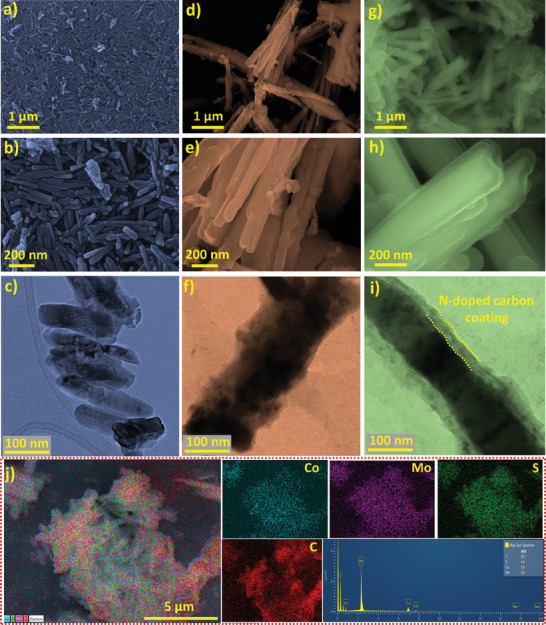
a,b) FE‐SEM images of the CoMoO_4_ sample. c) TEM images of the CoMoO_4_ sample. d,e) FE‐SEM images of the CoMoS sample. f) TEM images of the CoMoS sample. g‐h) FE‐SEM images of the CoMoS@NC sample. i) TEM images of the CoMoS@NC sample. j) EDS elemental color mapping and EDS elemental spectrum of CoMoS@NC sample.

**Figure 3:**
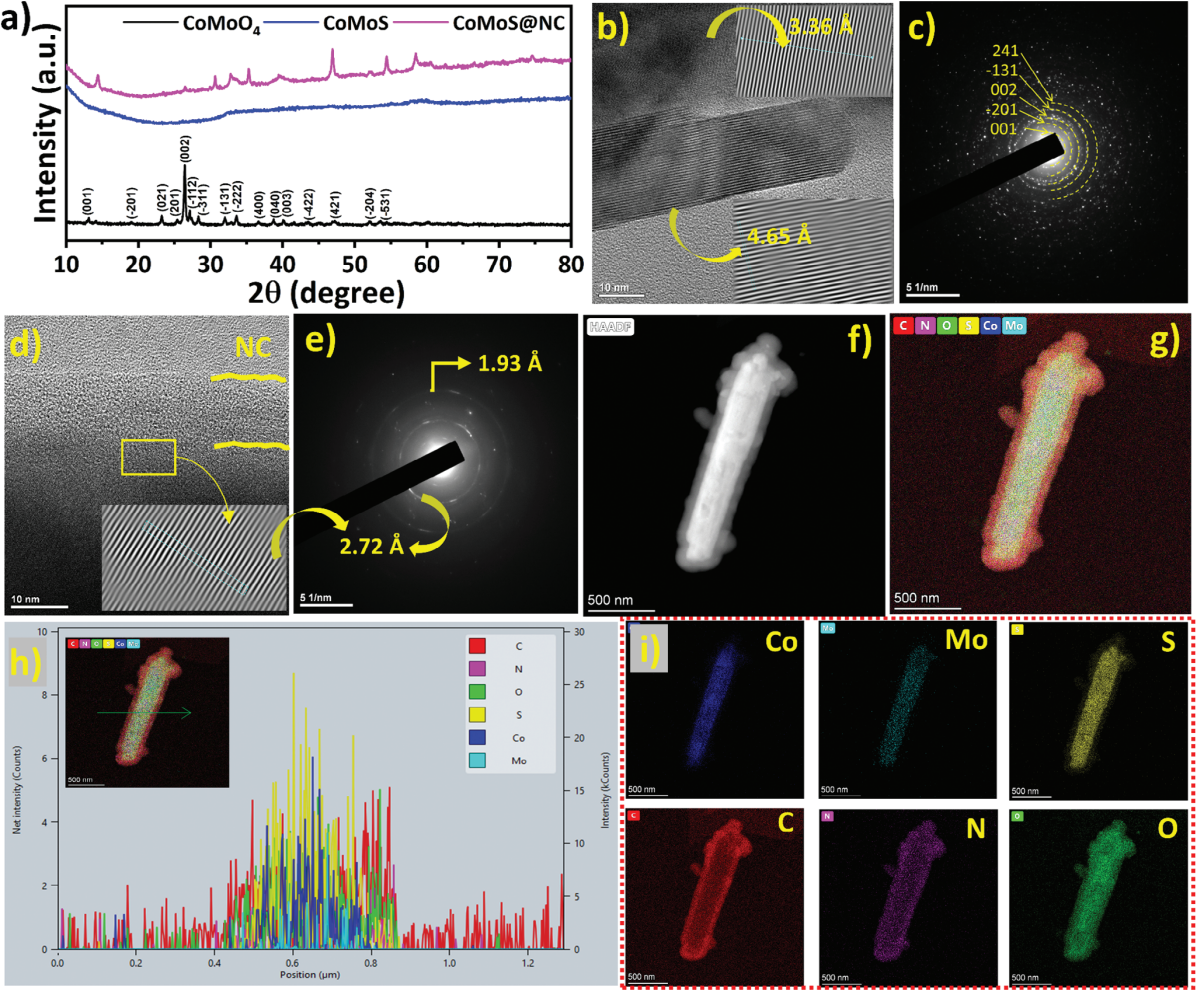
a) XRD patterns of different samples. b) HR‐TEM images of the CoMoO_4_ sample showing lattice fringes and c) corresponding SAED patterns. d) HR‐TEM image, and e) corresponding SAED patterns of the CoMoS@NC sample. f) HAADF‐STEM image. g) corresponding EDS color mapping. h) corresponding elemental line scan spectrum, and i) individual color spectrum images of different elements of the CoMoS@NC sample.

**Figure 4:**
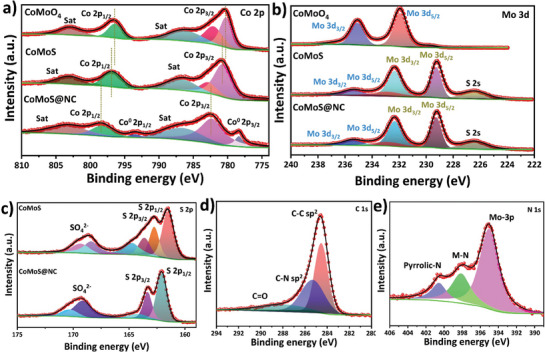
a) Comparative Co 2p core level XPS results of CoMoO_4_, CoMoS, and CoMoS@NC samples. b) Comparative Mo 3d core level XPS results of CoMoO_4_, CoMoS, and CoMoS@NC samples. c) Comparative S 2p core level XPS results of CoMoS and CoMoS@NC samples. d) C 1s core level XPS results of CoMoS@NC sample. e) N 1s core level XPS results of CoMoS@NC sample.

The experimental procedure to prepare the encapsulated composite material is schematically represented in **Figure** **1**. The detailed experimental process can be found in Section 1 of the electronic supplementary information (ESI) along with the relevant materials and methods that were implemented. Briefly, CoMoO_4_ was prepared with a hydrothermal method mainly to produce nanoparticles with a well‐defined homogenous morphology and a stoichiometric metal oxide composition that acted as a template for the subsequent sulfurization process. Our earlier work informed us that a stoichiometric metal oxide composition is highly crucial for optimal sulfurization and leads to improved electrochemical results.^[^ ^19^ ^]^ Then, CoMoS was synthesized in a second hydrothermal reaction. Hydrothermal sulfurization helps to counteract particle agglomeration and keeps the morphology of the nanoparticles intact to produce a stoichiometrically consistent metal sulfide as the final product owing to the homogeneous dispersion of the sulfur anions in the aqueous solution. Finally, the CoMoS was coated with polydopamine using dopamine hydrochloride as the precursor in a pH‐maintained aqueous solution with simple stirring at room temperature. The polydopamine shell was carbonized at high temperature to form the nitrogen‐doped carbon matrix around the CoMoS core, to further improve the electronic and ionic conductivity of the composite.The physical morphology of all the samples was analyzed with the aid of field emission scanning electron microscopy (FE‐SEM). Figures 2a and b show FE‐SEM images of the CoMoO_4_ sample, which is observed to consist of nanosized rod‐like structures. Higher magnification in **Figure** **2**b shows the uniformity of these structures. The average dimensions of each nanorod were ≈30 nm in diameter and 200 nm in length. Figure 2c shows the transmission electron microscope (TEM) image of the same sample and further highlights the morphological uniformity of the nanomaterial. Figure 2d,e shows FE‐SEM images of the CoMoS sample. Here, the nanoparticles can be seen to be slightly larger, most probably due to the additional energy provided by aqueous phase sulfurization, which would promote nanomaterial growth. Nonetheless, the rod‐like morphology continued to prevail, which, even though not to the same extent as the CoMoO_4_, still possesses a degree of homogeneity. This is a highly satisfactory result especially if compared with the high‐temperature solid‐state sulfurization process. The FE‐SEM images of high‐temperature sulfurization can be seen in ESI Figure S1 (Supporting Information), where agglomerated nonhomogeneous metal sulfide chunks are visible. The larger particle size and dissimilar morphology translate into fewer exposed active material sites for ion storage. Figure 2f shows a TEM image of a CoMoS nanorod. After the polydopamine coating step, it eventually came to light that the CoMoS particles seen in the FE‐SEM images in Figures 2d and e are rather loosely attached rods that can be quite easily separated into smaller nanorods by stirring them overnight in aqueous solution during the polydopamine coating process as seen in Figure 2g,h. CoMoS@NC exhibits superior structural homogeneity with all the nanorods covered with a rather pleasing translucent nitrogen‐doped carbon coating derived from polydopamine. Figure 2i shows the TEM image of a single CoMoS@NC nanorod, further verifying the existence of the nitrogen‐doped carbon coating over the CoMoS core. Figure 2j shows the energy‐dispersive spectroscopy (EDS) elemental color maps and elemental spectrum for the CoMoS@NC sample. All the relevant elements can be observed except nitrogen. This could be due to the very small amount of nitrogen present in the composite (< 2 atomic weight percent), coupled with the lower number of EDS channel detectors (1 detector) in this case. Importantly, the carbon color map suggests the quite significant presence of carbon, verifying that the N‐doped carbon coating had been successfully applied. As a control sample, the CoMoS was also prepared using a one‐step hydrothermal approach. In this method, Co, Mo, and S precursors were mixed to obtain the final sample, designated as CoMoS‐H. The physical morphology of CoMoS‐H is illustrated in Figure S2 (Supporting Information). The one‐step hydrothermal process resulted in the formation of nonhomogeneous metal sulfide nanoparticles, ranging from powder‐like material to large flakes. Carbonizing such nanomaterials with dopamine polymerization came with a major hurdle of too much carbon nanosphere agglomeration over the CoMoS core as shown in Figure S3 (Supporting Information). While nanospheres could also agglomerate in the case of CoMoS nanorods, most of these were easily decanted during the centrifuge process due to the relatively larger nanorods of CoMoS settling effectively during centrifugation. The unintended agglomeration of polydopamine nanospheres alongside the desired coating is a well‐known concern.^[^ ^20^ ^]^ The excessive deposition of carbon results in reduced cell capacity due to the lower specific capacity of carbon. Consequently, this method was not pursued further, leading us to choose two‐step hydrothermal sulfurization as an efficient alternative sulfurization method.The crystalline structure of the prepared nanocomposites was examined using X‐ray diffraction (XRD) analysis. As depicted in **Figure** **3**a, the XRD patterns of CoMoO_4_ closely resemble the standard XRD pattern (JCPDS No. 21–0868).^[^ ^21^ ^]^ The most prominent diffraction peaks at 26.45° can be attributed to the (002) crystal planes of monoclinic CoMoO_4_. The pattern reveals additional crystal planes corresponding to different 2*θ* values. Overall, the relatively sharp and well‐defined peaks indicate the favorable crystallinity of the CoMoO_4_ nanoparticles. Notably, no additional peaks are observed, indicating the high purity of the synthesized sample and the successful formation of the stoichiometric CoMoO_4_ template. Conversely, the XRD patterns of CoMoS and CoMoS@NC nanoparticles are also presented in the same figure. The absence of diffraction peaks in hydrothermally synthesized CoMoS can be attributed to the amorphous nature of the material formed during the synthesis process. It has been well‐documented that hydrothermal synthesis, often produces materials with low crystallinity, and that holds prominently true, especially in the case of cobalt molybdenum sulfide composites.^[^ ^22^ ^]^ In contrast, the CoMoS@NC sample demonstrates distinct diffraction peaks that precisely correspond with the CoMoS_3.1_ phase (JCPDS No. 16–0439).^[^ ^23^ ^]^ Calcination at 500 degrees induces a thermal treatment that promotes crystallization by removing residual water, and organic species/impurities, and promoting atomic rearrangement. This results in the formation of well‐defined crystalline structures, leading to the appearance of diffraction peaks in the XRD pattern.^[^ ^24^ ^]^ The observed diffraction peaks confirm the transition from an amorphous to a crystalline state, indicating the formation of a more ordered and structured CoMoS phase.^[^ ^25^ ^]^ The alkaline environment during dopamine treatment may also have contributed to crystallinity, as the pH of the reaction solution is well known to affect the crystallinity of the nanomaterial formed.^[^ ^26^ ^]^ However, further study on this seems out of the scope of the present work, mainly because the optimum and the most efficient polydopamine coating can be achieved at a pH of 8 to 8.5, rendering the variable pH study redundant.^[^ ^16^, ^27^ ^]^ In addition to the CoMoS_3.1_ peaks, the appearance of the carbon peak near 26.5° corresponds to the (002) lattice plane of carbon and again verifies its presence in the composite. Figure 3b shows high resolution transmission electron microscope (HR‐TEM) images of the CoMoO_4_ sample with lattice fringes corresponding to the (002) and (−201) crystal planes, and d‐spacings of 3.36 and 4.65 Å, respectively. Figure 3c depicts the corresponding selected area diffraction (SAED) patterns, which also have clearly distinguishable crystal diffraction patterns. Likewise, Figure 3d depicts the HR‐TEM image, and Figure 3e the corresponding SAED patterns of the CoMoS@NC sample. These results reflect the relatively milder crystalline nature of the composite compared to CoMoO_4_ and are highly consistent with the XRD results discussed earlier. Figure 3f shows high‐angle annular dark field scanning transmission electron microscopy (HAADF‐STEM) images of the CoMoS@NC sample, providing unequivocal evidence of the metal sulfide core and N‐doped carbon shell configuration. Figure 3g–i provides further support with elemental color mapping and line scan elemental analysis results. The results indicate the undoubted presence of Co, Mo, and S in the core, whereas the presence of C and N concentrated around the exterior of the nanorod confirms the existence of the N‐doped carbon shell. Oxygen is also present throughout the coated nanorod, seemingly evenly distributed between the metal oxide phase comprising the nanorod and the N‐doped carbon coating as a heteroatom.The chemical states of different samples were determined with X‐ray photoelectron spectroscopy (XPS) analysis. The XPS survey spectra of CoMoO_4_, CoMoS, and CoMoS@NC are presented in Figure S4 (Supporting Information). Likewise, **Figure** **4**a shows the comparative Co 2p core level spectra of the CoMoO_4_, CoMoS, and CoMoS@NC samples.^[^ ^28^ ^]^ The positive shift of the respective 2p_3/2_ and 2p_1/2_ peaks toward higher binding energy suggests a decrease in electron density as a result of sulfurization and the nitrogen‐doped carbon coating that covers the element Co. Apart from the most obvious Co 2p peaks, the Co^0^ 2p peaks observed in the case of CoMoS@NC are most probably due to the oxygen‐deficient carbonization as well as the reducing environment provided by the polydopamine coating. It has been reported that the catechol groups present in the polydopamine coating help reduce the metal cations.^[^ ^29^ ^]^ Likewise, Figure 4b shows the comparative core level spectra of Mo 3d along with low‐intensity S 2s peaks. For the CoMoO_4_ sample, the Mo 3d peaks were deconvoluted into Mo 3d_5/2_ and 3d_3/2_ of Mo^6+^, respectively,^[^ ^21^ ^]^ whereas the Mo 3d peaks of CoMoS and CoMoS@NC were deconvoluted into 3d_5/2_ and 3d_3/2_ of Mo^4+^, thereby verifying the presence of the Mo‐S bond.^[^ ^30^ ^]^ After undergoing sulfurization and carbonization processes, trace amounts of Mo^6+^ were observed in the Mo 3d_5/2_ and 3d_3/2_ peaks, likely attributed to the hydrothermal sulfurization conditions.^[^ ^31^ ^]^ Along with the Mo 3d peaks, a low‐intensity S 2s peak, originating from the solitary sulfur deposits, can be observed on the spectra of CoMoS and CoMoS@NC. Figure 4c shows the comparative S 2p core level spectra of the CoMoS and CoMoS@NC samples. The S 2p spectrum of both samples can be deconvoluted mainly to S 2p_1/2_ and S 2p_3/2_ doublets, indicating the metal sulfur bonds. The XPS peaks observed at binding energies of 163.68 eV and 164.78 eV in the CoMoS sample are attributed to the presence of thiol groups. The presence of these peaks suggests the incorporation of thiol‐containing ligands, possibly originating from the thioacetamide precursor used in the hydrothermal synthesis of CoMoS. Likewise, the broad peak at higher BE (168 eV) is associated with superficial sulfur oxidation with a high valance state of the metal‐sulfur bond.^[^ ^32^ ^]^ Furthermore, the positive shift of all the peaks toward higher BE for CoMoS@NC suggests the decrease in the electron cloud associated with sulfur species mainly because of the nitrogen‐doped carbon layer that covers the metal sulfide nanostructure. Likewise, the C 1s spectrum in Figure 4d is deconvoluted into three peaks corresponding to the C─C sp^2^, C─N sp^2^, and C = O groups of the CoMoS@NC sample, thereby verifying the graphene‐like nitrogen‐doped carbon coating covering the metal sulfide nanostructure.^[^ ^33^ ^]^ The presence of nitrogen in the carbon coating is verified by the N 1s XPS core level spectrum as presented in Figure 4e. The N 1s spectrum overlaps with the Mo 3p spectrum and the deconvoluted peaks correspond to the Mo 3p_3/2_ at BE of 395 eV, metal nitride bond (Mo‐N) at BE of 397.7 eV, and pyrrolic nitrogen at BE of 400.5 eV, and highlights the presence of nitrogen as a dopant in the carbon lattice.^[^ ^34^ ^]^ While the oxidized sulfur may impose many adverse effects on the overall performance of the battery system, the nitrogen‐doped carbon coating over the CoMoS core serves as a protective layer, minimizing further sulfur oxidation and maintaining active material integrity. Nitrogen doping enhances electrical conductivity, reducing charge transfer resistance and improving rate capability in the LIB half‐cell. Additionally, the coating acts as a physical barrier, confining polysulfides within the core, suppressing the shuttle effect, and enhancing coulombic efficiency while mitigating capacity loss over cycling.^[^ ^35^ ^]^

### 2.2 Electrochemical Characterization

**Figure 5:**
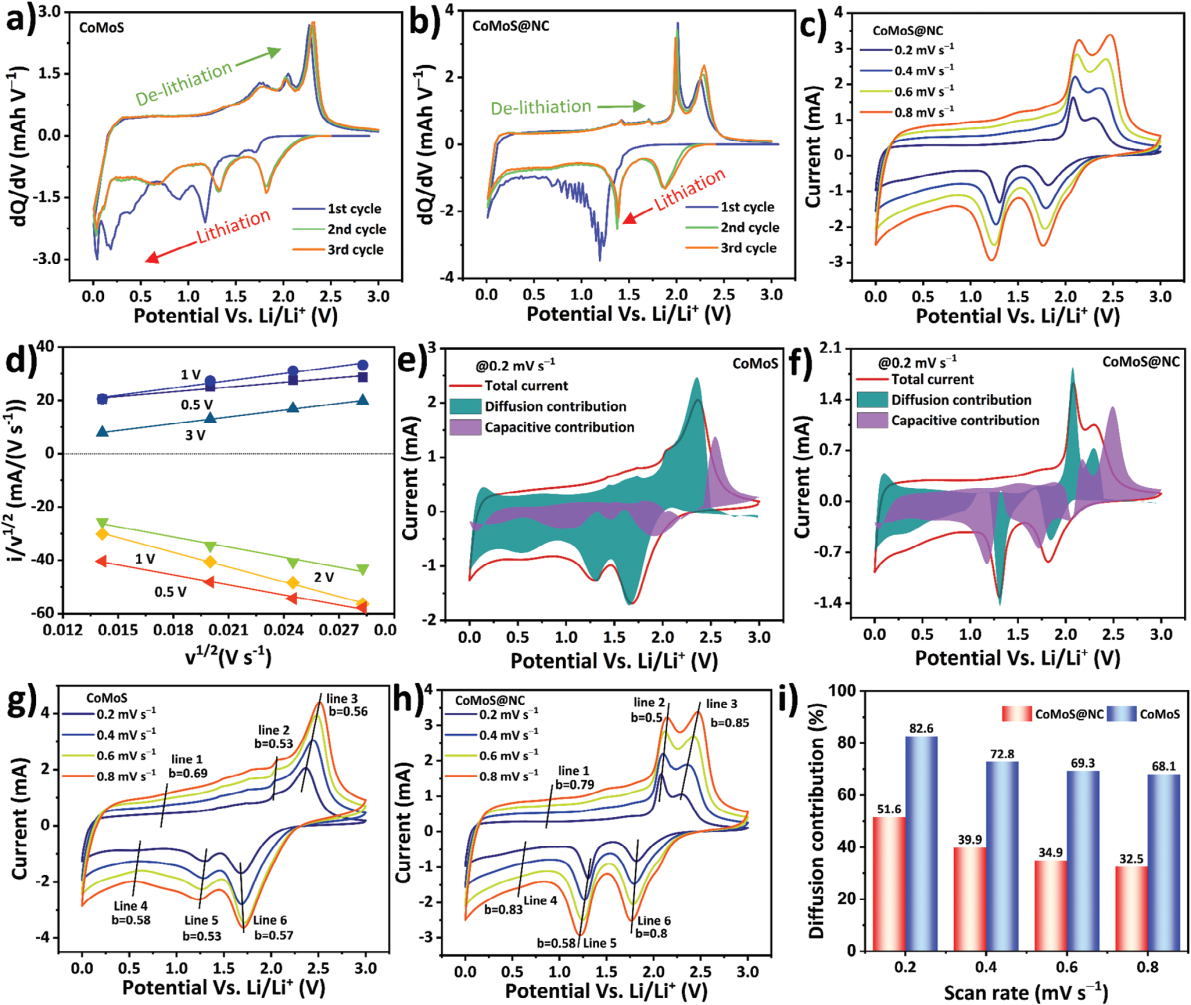
a) *dQ*/*dV* plot of the CoMoS cell. b) *dQ*/*dV* plot of the CoMoS@NC cell. c) CV curves over different scan rates for CoMoS@NC cell. d) Linear fitting of various anodic and cathodic scans of CoMoS@NC CV. Diffusion‐controlled and capacitive contributions plot compared to total current at 0.2 mV s⁻^1^ for e) CoMoS and f) CoMoS@NC cell. b‐value calculation at several places for g) CoMoS and h) CoMoS@NC cell. i) Diffusion‐controlled contribution bar diagram for CoMoS and CoMoS@NC cells at different scan rates.

The materials and methods pertaining to the electrochemical characterization are presented in section 2.2 of the ESI. Briefly, CR‐2032 half‐cells with CoMoS or CoMoS@NC as the working electrode and lithium foil as the counter and reference electrode were assembled inside an argon‐filled glove box. All cells were checked to ensure the open‐circuit voltage was satisfactory (≈3 V). Several charge‐discharge cycles were run for each cell at a constant current density of 200 mA g^−1^. Charge‐discharge cycles at different current densities ranging from 100 mA g^−1^ to 1000 mA g^−1^ were performed as a rate capability test. Differential capacity (*dQ*/*dV*) curves were plotted to identify the electrochemical charge storage kinetics and relevant reactions inside the respective coin cells. The *dQ*/*dV* analysis intensifies and enhances the resolution of each electrochemically induced peak, particularly when compared to CV analysis.^[^ ^36^ ^]^ **Figure** **5**a shows the *dQ*/*dV* plot of the CoMoS cell. The plot was derived from the constant current charge‐discharge cycles run at the current density of 200 mA g^−1^. The first lithiation cycle shows several pronounced peaks namely at 1.7, 1.5, 1.2, 0.9, 0.4, and 0.2 V. The peaks ranging from 1.7 to 0.4 V are assigned to the reduction of Co and Mo ions to their respective metallic phases owing to the Li^+^ insertion reaction inside the CoMoS lattice.^[^ ^15^ ^]^ The peaks ranging from 0.4 V to the cutoff potential can be assigned to the further reduction of Li*_x_* CoMoS_3.13_ to metallic Co and Mo as well as the formation of the solid electrolyte interphase (SEI) layer.^[^ ^14^, ^15^, ^37^ ^]^ The possible lithiation reaction can be expressed as follows:^[^ ^14^ ^]^

(1) $${\mathrm{CoMoS}}_{3.13}+x{\mathrm{Li}}^{+}+xe^{-}\to {\mathrm{Li}}_{x}{\mathrm{CoMoS}}_{3.13}$$

(2) $${\mathrm{Li}}_{x}{\mathrm{CoMoS}}_{3.13}+6.26Li^{+}+6.26e^{-}\to 3.13Li_{2}S+\mathrm{Mo}+\mathrm{Co}$$

The subsequent de‐lithiation cycle is composed of three distinct peaks at 1.7, 2.0, and 2.3 V which can be ascribed to the oxidation of Co and Mo and the formation of CoS*_x_* and MoS_2_ as governed by the following equations:

(3) $${\mathrm{Li}}_{2}S+\mathrm{Mo}\to {\mathrm{MoS}}_{2}+4Li^{+}+4e^{-}$$

(4) $$x{\mathrm{Li}}_{2}S+\mathrm{Co}\to {\mathrm{CoS}}_{x}+2x{\mathrm{Li}}^{+}+2xe^{-}$$

The next lithiation cycle and those from thereon forward have two distinctive peaks at 1.8 and 1.3 V, which can be attributed to the intercalation of Li^+^ into the MoS_2_ and CoS*_x_* matrix whereas the broad peak at 0.6 V corresponds to the conversion reaction of Li^+^ with metallic Co and Mo. The *dQ*/*dV* plot of CoMoS@NC as shown in Figure 5b exhibits all the peaks associated with the major oxidation and reduction reactions, but other smaller peaks seem to be suppressed most probably owing to the carbon layer covering the CoMoS. Furthermore, the repeatability of lithiation and de‐lithiation over the second and third cycles for both CoMoS and CoMoS@NC suggest the electrochemical stability of the fabricated materials. Cyclic voltammetry (CV) was performed to analyze the charge storage kinetics of the fabricated cells. Figure 5c shows the CV curves of CoMoS@NC cells recorded at different scan rates ranging from 0.2 mV s^−1^ to 0.8 mV s^−1^. The CV oxidation and reduction peaks for CoMoS (Figure S5, Supporting Information) and CoMoS@NC are both analogous to the *dQ*/*dV* plot and suggest the good reproducibility of the work. The increase in current response as well as the absence of the appearance of any foreign peak with the increased scan rate suggests the good reversibility of the reaction kinetics of the fabricated material. The absence of exotic peaks is expected from the well‐synthesized materials and a well‐made cell, indicating that the electrochemical system aligns with known reactions (Equations (1), (2), (3), (4)), ruling out unexpected processes. Peak shifts result from electrode surface reaction kinetics and time‐dependent behavior. Specifically, an anodic peak shifting right suggests slower oxidation, requiring a higher potential. Conversely, a left‐shifted cathodic peak implies faster, more kinetically controlled reduction at higher scan rates. The comparison of the CV with the mass‐specific current response for CoMoS and CoMoS@NC revealed an increased CV area for CoMoS@NC, as shown in Figure S6 (Supporting Information). This suggests its higher charge storage capability, most definitely owing to the synergistic contribution of CoMoS and the nitrogen‐doped carbon matrix. Hence, it is important to understand and quantify the charge storage contributions to determine whether charge storage is attributable to the diffusion‐controlled contribution from the conversion/intercalation reaction on the metal sulfide lattices or the surface adsorption on the nitrogen‐doped carbon matrix.

The total capacity of CoMoS and CoMoS@NC is determined by analyzing the contributions of surface‐controlled charge storage and diffusion‐controlled charge storage, as calculated using equation (5):^[^ ^38^ ^]^

(5) $$i\left(v\right)=k_{1}v+k_{2}v^{\frac{1}{2}}$$

where *i* is the total current, *k*_1_ *v* is a capacitive contribution, and $k_{2}v^{\frac{1}{2}}$ is a diffusion‐controlled contribution with *v* as the scan rate. Rearranging Equation (5) gives:

(6) $$\frac{i(v)}{v^{\frac{1}{2}}}=k_{1}v^{\frac{1}{2}}+k_{2}$$

Determination of the slope and intercept of the line described by Equation (6) enables *k*_1_and *k*_2_, the capacitive and diffusive contributions, respectively, to be determined. Figure 5d shows the linear fittings of various anodic and cathodic CV scans of CoMoS@NC. The diffusion‐controlled and capacitive contributions are plotted using the resulting intercept and slope obtained from Figure 5d, as shown in Figure 5e for CoMoS and Figure 5f for CoMoS@NC, at a scan rate of 0.2 mV s^–1^. Clearly evident is that the diffusion contribution is dominant for the CoMoS cell, whereas a substantial increment in the capacitive contribution is observed for the CoMoS@NC cell. This increment for CoMoS@NC supposedly originates from the nitrogen‐doped carbon matrix that encapsulates the CoMoS nanostructure.^[^ ^39^ ^]^ The charge storage contribution can be cross‐verified using the equation popularly known as the power law:

(7) $$i=av^{b}$$

where a and b are adjustable parameters, *v* is the scan rate (mV s^–1^), and *i* is the current (A). Equation (7) can be simplified to,

(8) log(i)=blog(v)+log(a)

**Figure 6:**
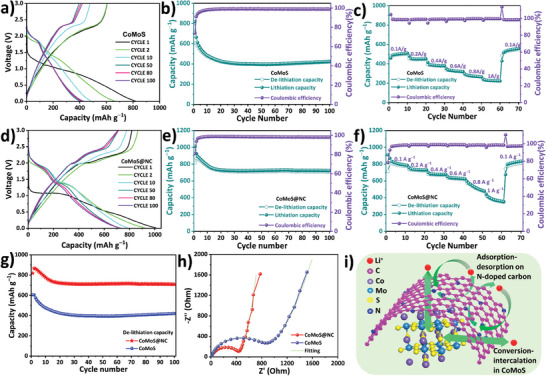
a) Lithiation/de‐lithiation cycles of the CoMoS cell. b) Lithiation/de‐lithiation capacities and coulombic efficiency of CoMoS cell over 100 cycles. c) Rate capability test of CoMoS cell. d) Lithiation/de‐lithiation cycles of the CoMoS@NC cell. e) Lithiation/de‐lithiation capacities and coulombic efficiency of CoMoS@NC cell over 100 cycles. f) Rate capability test of CoMoS@NC cell. g) De‐lithiation capacity comparison of CoMoS and CoMoS@NC cells. h) EIS comparison of CoMoS and CoMoS@NC cells. i) Artistic representation of charge storage kinetics for CoMoS@NC cell.

Specifically, the value of b determines the nature of charge storage, with a b‐value of 1 indicating an ideal surface adsorption‐controlled contribution and a b‐value of 0.5 indicating an ideal diffusion‐controlled contribution.^[^ ^38^ ^]^ For well‐homogenized results, the b‐value was calculated at several positions on the CV curves as represented by the line numbers shown in Figure 5g,h. Governed by Equation (8), the slope of the linear plot of log(i) versus log(v), as shown in Figure S7 (Supporting Information), yields the value of b. The *b*‐values are in good agreement with the diffusion and capacitive contribution curves. Particularly, when comparing the peaks that stood in drastic contrast, such as those of CoMoS ≈2.3 V on the anodic scan, the capacitive contribution is undetectable and the diffusive contribution is maximized, resulting in a b‐value of 0.56. Similarly, for CoMoS@NC the capacitive contribution is negligible on the anodic scan at 2 V and the cathodic scan at 1.3 V, which are perfectly complimented by b‐values of 0.5 and 0.58, respectively. Finally, the diffusion contribution for both electrodes was plotted for different scan rates ranging from 0.2 to 0.8 mV s^−1^ as shown in Figure 5i. This comparison indicates that the CoMoS@NC has a much smaller diffusion contribution as compared to CoMoS mainly due to the presence of the nitrogen‐doped carbon outer layer that promotes charge storage controlled by surface adsorption. This is more apparent toward higher scan rates: as the scan rate increases, the diffusion contribution decreases more rapidly for CoMoS@NC as compared to CoMoS. This implies that an increasing scan rate continually shortens the time required for the lithium ions to diffuse into the CoMoS lattice. In contrast, the lithium ions are adsorbed onto the abundantly present carbon outer layer, thereby progressively increasing the capacitive nature of charge storage. The charge storage in CoMoS and CoMoS@NC was quantitatively analyzed by constant current charge‐discharge cycling within the potential range 0.05−3 V in a half‐cell arrangement with lithium foil as the counter and reference electrode. **Figure** **6**a shows the representative lithiation/de‐lithiation cycles of the CoMoS cell that was run for 100 cycles at a current density of 0.2 A g⁻^1^. As seen in the figure, the first lithiation and de‐lithiation capacities for the CoMoS cell are registered as 818 and 605.88 mAh g⁻^1^, respectively, whereas for the second cycle, the respective lithiation and de‐lithiation capacities are 664.45 and 605.18 mAh g⁻^1^. The coulombic efficiency for the first and second cycles are 74% and 91%, respectively. The irreversible capacity loss in the 1st and 2nd cycles is attributed to the formation of a SEI layer. The lithiation and de‐lithiation capacities from the 3rd cycle onward are almost similar and a coulombic efficiency of almost 100% was registered up to the 100th cycle as depicted more clearly in Figure 6b. After constant capacity fading up to approximately the 25th cycle, the lithiation and de‐lithiation capacities remained unchanged until the 100th cycle. The lithiation and de‐lithiation at the 100th cycle are 425.22 and 420.23 mAh g⁻^1^, respectively, indicating capacity retention of ≈70%. Likewise, Figure 6c shows the specific capacities of CoMoS cells with different current densities of 0.1, 0.2, 0.4, 0.6, 0.8, and 1 A g⁻^1^, and back to 0.1 A g⁻^1^. The de‐lithiation capacity registered at the end of each current density cycle is 503, 449.2, 378.1, 318.8, 264.3, and 222.6 mAh g⁻^1^ and back to 549.3 mAh g⁻^1^. Despite the smaller specific capacity, the CoMoS cell performed well under different current densities to ultimately return to its original value.

The lithiation/de‐lithiation profile of the CoMoS@NC cell can be seen in Figure 6d, from which it is clear that the lithiation/de‐lithiation capacities of this cell greatly improved compared to that of the CoMoS cell. Specifically, for the 1st cycle, these capacities are 1010.25 and 818.21 mAh g⁻^1^, and for the 2nd cycle 924.22, and 866 mAh g⁻^1^, respectively. In addition to the obvious increase in the specific capacity, the performance of the CoMoS@NC cell was superior in other ways such as the substantially increased coulombic efficiency of 81% and 93.74% in the 1st and 2nd cycles, respectively. The higher the coulombic efficiency in the starting cycles, the lesser the material loss on the SEI layer, and the higher the specific capacity becomes in subsequent cycles. Figure 6e shows the coulombic efficiency and specific capacities of the CoMoS@NC cell for 100 cycles ran at a current density of 0.2 A g⁻^1^. Capacity fading ceased even before the 20^th^ cycle, unlike that of the CoMoS cell, and remained unchanged until the 100th cycle. The lithiation and de‐lithiation capacities of the 100th cycle are 723.12 and 709.06 mAh g⁻^1^, respectively, which is almost the same as that of the 20^th^ cycle and constitutes capacity retention of approximately 87% compared to that of the first cycle, a marked improvement compared to CoMoS. The consistency and absence of any sudden change in the specific capacity suggest the excellent reversible cyclability of the cell.

The CoMoS@NC cell maintained its high specific capacity throughout the different current densities ranging from 0.1 A g⁻^1^ to 1 A g⁻^1^, as depicted in Figure 6f. The respective de‐lithiation capacities registered at the end of each current density cycle are 773.8, 722.1, 669.1, 616.5, 472.4, and 348.1 mAh g⁻^1^, and back to 816.7 mAh g⁻^1^. The specific capacities for CoMoS@NC at the end of each current density cycle are almost twice as high as those of the CoMoS cells. Furthermore, the capacity retention that accompanied the reversal of the current density to 1 A g⁻^1^, without any loss in the capacity, attests to the excellent capacity retention ability and reversibility of the cell. Figure 6g shows a distinct comparison of the de‐lithiation capacity of CoMoS and CoMoS@NC at a current density of 0.2 A g⁻^1^ up to 100 cycles. The CoMoS@NC leads the specific de‐lithiation capacity by a margin of almost 300 mAh g⁻^1^ compared to that of CoMoS. The increased capacity mainly arises from the nitrogen‐doped carbon matrix. The defect‐rich carbon matrix, promoted by the functional groups, facilitates the storage of additional charge by surface adsorption phenomena as verified by the kinetic study presented earlier. Furthermore, the encapsulation of pristine CoMoS with the carbon matrix increases the charge transfer conductivity and also provides a buffer against repetitive volumetric expansion and compression during the lithiation and de‐lithiation processes, respectively. The improved conductivity of CoMoS@NC compared to that of CoMoS was also verified by EIS analysis as shown in the Nyquist plot in Figure 6h. The smaller semicircle in the higher frequency region for CoMoS@NC suggests that its charge transfer resistance is lower than that of CoMoS. The fitted equivalent electrical circuit and respective parameters are shown in ESI Figure S8 (Supporting Information). Figure 6i shows an artistic representation of the charge storage kinetics for CoMoS@NC. Here, the nitrogen‐doped carbon shell stores the charge on its surface and defect sites through physical adsorption. In contrast, the lithium ions can migrate through the defect sites as well as the hexagonal carbon mesh to participate in the conversion/intercalation reaction in the CoMoS lattice as governed by the oxidation‐reduction reactions presented earlier. These ion transport and charge storage processes synergistically contribute toward the superior performance of the CoMoS@NC composite as an active material of LIBs.

**Figure 7:**
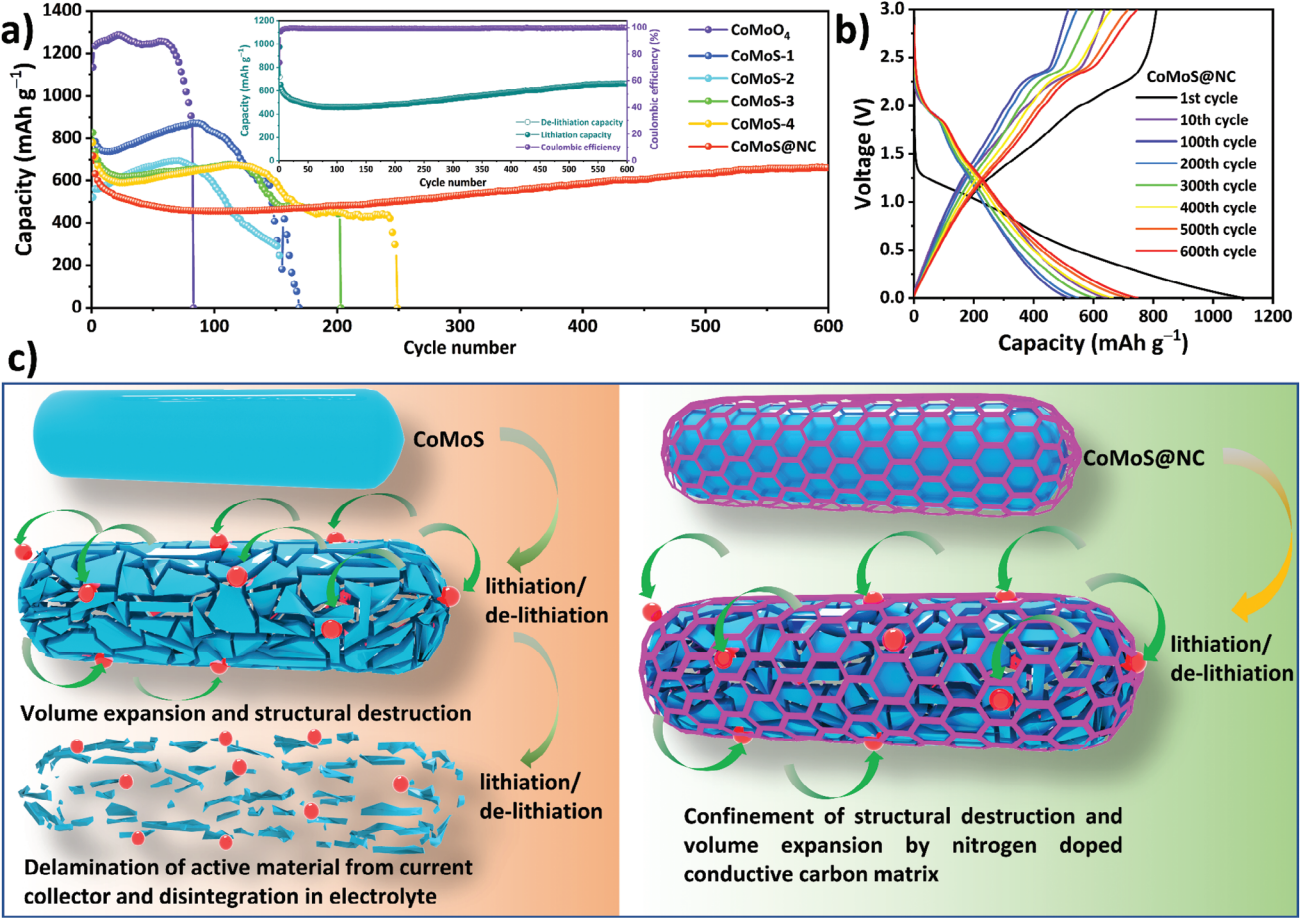
a) Long‐term stability test showing de‐lithiation capacities of different cells (inset: lithiation/de‐lithiation capacities and corresponding coulombic efficiency of CoMoS@NC cell). b) Selected lithiation/de‐lithiation capacity cycles of CoMoS@NC cell of 600 cycles. c) Artistic representation of lithium insertion and volume expansion induced structural destruction and role of N‐doped carbon shell to mitigate that.

The effect of the nitrogen‐doped carbon coating on the stability of the cell was monitored by conducting a long‐term stability test of several cells with various anodic compositions, as shown in **Figure** **7**a. The weight ratio of sulfur:CoMoO_4_ (S:M) was varied in an attempt to optimize the composition of the anode for the next step of N‐doped carbon coating. As seen in Figure 7a, despite the initially high capacity of the pristine CoMoO_4_ electrode, its stability is severely compromised by repetitive lithiation/de‐lithiation cycling, resulting in the cell completely collapsing ≈ 50 cycles. In comparison, the stability of the cells with CoMoS‐1 and CoMoS‐2, prepared with S:M mass ratios of 1 and 2, respectively, is slightly higher than that of CoMoO_4_, although capacity fading started at ≈80 cycles. Contrary to this, CoMoS‐3 and CoMoS‐4 (S:M mass ratios of 3 and 4) had good stability for up to 150 cycles with reasonable capacity. Hence CoMoS‐3 was selected for N‐doped carbon coating for subsequent testing (and named CoMoS throughout the paper). As for the CoMoS@NC, it shows remarkable cycling stability of 600 cycles at a current density of 0.5 A g^−1^, after which the de‐lithiation capacity of CoMoS@NC remained at 661.4 mAh g⁻^1^ for a capacity retention rate of 92% compared to the initial de‐lithiation capacity. Interestingly, the capacity tended to increase slightly with increasing cycling (i.e., as the number of cycles increased), which is most probably due to the activation of additional electroactive sites and improved electrolyte diffusion as the cycling progressed.^[^ ^40^ ^]^ Similarly, the observed decrease in capacity before 100 cycles during long‐term stability testing can be attributed to the initial formation of the SEI layer on the electrode surface. This process, essential for stable battery performance, temporarily reduces capacity. Subsequently, as the SEI stabilizes and the system undergoes activation, capacity tends to recover. The inset in Figure 7a displays the lithiation/de‐lithiation capacity of CoMoS@NC, along with the corresponding coulombic efficiency, over 600 cycles. The remarkable stability of the active material is evident from the sustained coulombic efficiency of 99.93% even after 600 cycles. Additionally, Figure 7b exhibits multiple lithiation/de‐lithiation cycles that demonstrate consistent oxidation/reduction plateaus, emphasizing the persisting stability of CoMoS@NC throughout the entire 600 cycles. The role of the carbon matrix in the excellent electrochemical performance of the CoMoS@NC is highlighted in the graphical depiction shown in Figure 7c. During the repetitive process of lithium insertion and extraction, the continuous changes in volume experienced by CoMoS render it vulnerable to structural damage. Consequently, the active material becomes delaminated from the current collector and eventually disintegrates or dissolves in the electrolyte. This disintegration of the active material leads to a rapid decline in capacity, as previously observed for the CoMoS samples. In contrast, CoMoS@NC exhibits different behavior. The N‐doped carbon matrix surrounding the CoMoS acts as a protective barrier, effectively mitigating the volumetric changes and preventing the disintegrated active materials from becoming detached from the current collector and dissolving in the electrolyte. As a result, the stability of the active material is significantly enhanced over numerous cycles. Moreover, TEM analysis was performed on the material collected from the cell after 600 cycles of stability test, and the results are quite consistent with the hypothesis proposed using Figure 7c. As can be seen in the TEM, HRTEM, and EDS images in the ESI Figure S9 (Supporting Information), the carbon shell seems still intact. While the nanoparticles generated from repeated lithiation, de‐lithiation induced CoMoS core disintegration, still inside the carbon shell. This confirms that the carbon shell has maintained its integrity throughout the stability test. **Table** **1** comprehensively highlights the superiority/comparability of the CoMoS@NC performance when compared with the relatable metal sulfide/carbon composite electrode materials as a LIBs anode half cell. As we can see, the CoMoS@NC with relatively easier and scalable synthesis techniques shows commendable and well‐balanced performance in capacity and stability front, at highly comparable current density.

**Table 1:** Comprehensive performance comparison of CoMoS@NC with similar electrode materials.

Electrode Materials	Current density [A g^−1]^	Stability [Cycles]	Final capacity [mAh g^−1^]	Reference
Co_3_S_4_/CoMo_2_S_4_@rGO	0.2	100	408.8	[15]
CoMoS_3.13_@ADC	0.2	150	986	[14]
CoMoS@C	0.5	200	715	[37]
CoMoO_3_@c‐CNFs	0.5	360	706	[41]
CoMoO_3_/C−S	1	500	979.6	[42]
Co_3_S_4_@C@MoS_2_	0.2	200	672.6	[43]
MoS_2_/CoMo_2_S_4_/Co_3_S_4_@graphene	2	600	437	[44]
Co_9_S_8_@MoS_2_	1	300	1048	[45]
CoS_2_@NCNTs‐650	0.5	200	1057.04	[46]
CoS_2_@NC@CNC yolk‐shell	1	100	641	[47]
CoS_2_–MnS@rGO	1	100	927	[48]
CoMoO_4_@C	0.5	100	790	[49]
3D Co_1−_ *_x_*_S_/MoS_2_	1	500	715	[50]
MoS_2_‐Co_3_S_4_	0.1	200	880	[51]
CoS/NC@MoS_2_ Hollow Spheres	1	400	802.4	[52]
Si/CoMo@NCP	1	400	745	[53]
Co‐MoS_2_/CC	2	300	611	[54]
MNCO‐500	0.1	200	1360	[55]
CoMoO_4_–CoO/S@rGO	2	300	700	[56]
CoMoS@NC	0.5	600	661.4	This work

**Figure 8:**
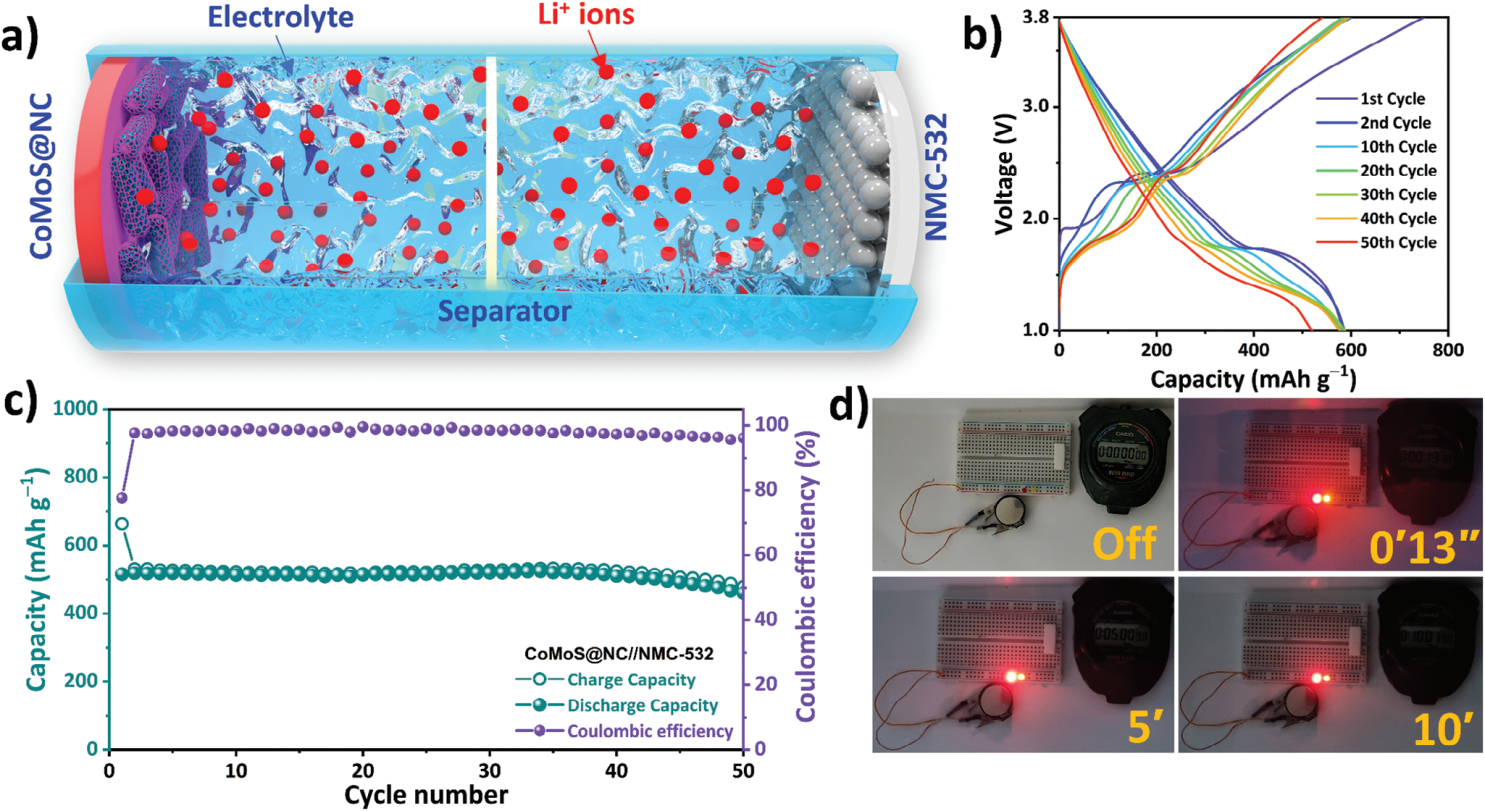
a) Full cell schematics with CoMoS@NC anode and NMC‐532 cathode. b) Charge/discharge cycles of full cell. c) Charge–discharge capacities and corresponding coulombic efficiency of the full cell up to 50 cycles. d) practical application of a fabricated cell.

Finally, the CoMoS@NC was used as the anode to fabricate a full cell with LiNi_0.5_Mn_0.3_Co_0.2_O (NMC‐532) as the cathode, as graphically depicted in **Figure** **8**a. Figure S10a (Supporting Information) shows the discharge capacity of the cathodic and anodic materials, which were subsequently used for mass balancing to fabricate the full cell. In addition, different anode‐to‐cathode (A:C) mass ratios were also assessed for optimization purposes. The discharge capacity of the cells with different A:C mass ratios is shown in Figure S10b (Supporting Information). Figure 8b shows the charge‐discharge curves of the as‐fabricated full cell within the potential window of 1−3.8 V cycled at a current density of 0.1 A g⁻^1^. The recurring charge‐discharge plateaus with minimal capacity fading suggest that the fabricated device is acceptably stable for practical applications. Figure 8c illustrates the sustained stability of the cell, demonstrated by its discharge capacities of 515.7 mAh g⁻^1^ in the 1st cycle and 460.2 mAh g⁻^1^ in the 50^th^ cycle. This indicates a remarkable capacity retention rate of ≈90%. Additionally, the cell exhibits notable coulombic efficiencies, with values of 77.7%, 97.7%, and 96.1% recorded in the 1st, 2nd, and 50th cycles, respectively. Finally, a fabricated full cell was used as a power source to run simulated loads to demonstrate its practicability. Figure 8d shows different colored LEDs lit up with one full cell for an extended period with minimal change in brightness. These demonstrations and electrochemical results confirm the appropriateness of CoMoS@NC as an advanced alternative anodic material for LIBs.

### 2.3 Computational Studies

The computational studies mentioned were conducted using Material Studio 2020 software. The optimization of structures was performed using the CASTEP code, employing the Perdew‐Burke‐Ernzerhof (PBE) functional for the exchange‐correlation energy and the Norm‐conserving optimized Troullier‐Martins (OTFG) pseudopotentials. The medium orbital cutoff quality used for the optimization was set at 1251.7 eV. The computational studies are performed on structures formed after the first lithiation/de‐lithiation cycles as governed by equation 4 from earlier. The initial geometry of CoS_2_ was obtained from the single crystal database. The carbon shell is assumed to be composed of graphene layers as suggested by earlier research.^[^ ^17^ ^]^ To simulate a structure where CoS_2_ is encapsulated by graphene, single and double graphene layers were added to the CoS_2_ geometry. The resulting structures were then subjected to full cell optimization, with a fixed basis set quality. The adsorption energy (*E*_ads_) for the process depicted in equation 9 was calculated using the equation: *E*_ads_ = *E*_CoS_ *_x_*_@C@Li_ − *E*_CoS_ *_x_*_@C_ − *E*_Li_. In this equation, *E*_CoS_ *_x_*_@C@Li_ represents the total energy of the system with CoS*_x_* encapsulated by graphene and Li adsorbed, *E*_CoS_ *_x_*_@C_ represents the total energy of the system with CoS*_x_* encapsulated by graphene, and E_Li_ represents the total energy of the lithium solid‐state structure. The difference in these energies gives the adsorption energy for the process.

(9) $${\;\;\mathrm{CoS}}_{x}@C+Li_{\mathrm{solid}}\overset{\Delta E_{\mathrm{ads}}}{\to }{\mathrm{CoS}}_{x}@C@\mathrm{Li}$$

**Scheme 1:**
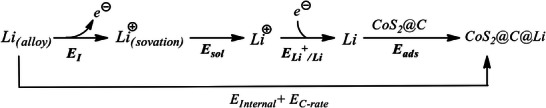
Lithium transfer and adsorption process during lithiation.

The lithium transfer process during lithiation potential from the alloy to CoS*_x_* @C can be summarized into four steps, each associated with its respective energy (**Scheme** **1**):

1. Ionization (E_I_): This step involves the removal of an electron from the lithium species in the alloy, resulting in the formation of Li^+^ ions. The energy associated with this ionization process is denoted as E_I_.
2. De‐solvation (E_Sol_): After ionization, the solvation shell surrounding the Li^+^ ions needs to be stripped away for further transfer. This de‐solvation step, represented by the energy E_Sol_, involves breaking the interactions between the Li^+^ ions and the surrounding solvent molecules. In this study, the process as depicted by equation (10) is employed to simulate the effect of the ethyl carbonate and dimethyl carbonate solvents in a 1:1 ratio. This simulation allows us to investigate the influence of the solvent mixture.

(10)

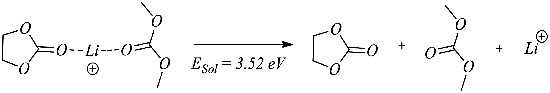

3. Lithium reduction (E_Li_^+^_/Li_): Once the Li^+^ ions are de‐solvated, they undergo a reduction reaction to form lithium metal (Li) at the surface of the CoS*_x_* @C material. This is a reduction process, which involves the gain of electrons by the Li^+^ ions.
4. Adsorption (E_ads_): In the final step, the lithium metal formed during the reduction process gets adsorbed onto the CoS*_x_* @C surface. The energy associated with this adsorption step represents the energy interaction between lithium and the CoS*_x_* @C material.

The total energy of the whole process is the sum of internal energy E_Li‐transfer_ and the external lithiation energy E_C‐rate_:

(11) $$E_{\mathrm{Li}-\mathrm{transfer}}+E_{C-\mathrm{rate}}=E_{I}+E_{\mathrm{sol}}+E_{\mathrm{Li}+/\mathrm{Li}}+E_{\mathrm{ads}}$$

(12) $$E_{\mathrm{Li}-\mathrm{transfer}}=\left(E_{I}+E_{\mathrm{Li}+/\mathrm{Li}}-E_{C-\mathrm{rate}}\right)+E_{\mathrm{ads}}+E_{\mathrm{sol}}$$

In this study, an approximation is utilized, assuming that the ionization energy (E_I_) and reduction energy (E_Li_^+^_/Li_) can be effectively offset by the energy associated with the applied lithiation rate (E_C‐rate_): $E_{I}+E_{{\mathrm{Li}}^{+}/\mathrm{Li}}\;-\;E_{C-\mathrm{rate}}=\;0$. This approximation implies that the net energy change during the transfer of lithium can be considered negligible, under the assumption that the energy needed for ionization and reduction is counterbalanced by the energy supplied by the applied lithiation potential.

#### 2.3.1 Discussion

The mechanism of LIB operation is complex, involving multiple processes. Encapsulating materials with graphene‐like carbonaceous networks can offer enhancements in various aspects, such as increased mechanical stability, the availability of active sites, and accelerated lithium transport.^[^ ^57^ ^]^ In this study, we investigate the electrokinetic enhancement provided by graphene on CoS*_x_* which is generated during the lithiation process.^[^ ^58^ ^]^ The lithiation process consists of two sequential steps: lithium transfer and adsorption into CoS*_x_* @C, forming CoS*_x_* @C@Li, and subsequent production of lithium ions, as described below:

(13) CoSx@C+LisolidΔELi−transfer⟶CoSx@C@Li→CoSx@C+Li++e+

**Figure 9:**
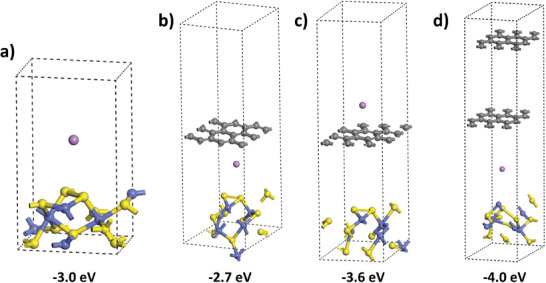
Location‐dependent adsorption energies of lithium on CoS*_x_* @C: a) without graphene layer, b) below single graphene layer, c) above single graphene layer, and d) below double graphene layers.

DFT calculations demonstrate that graphene facilitates the interaction between lithium alloy and CoS*_x_*, thereby increasing the overall efficiency of lithiation. The lithium energy transfer on the CoS_2_ surface is −3.0 eV (**Figure** **9**a). Comparatively, the different locations of lithium relative to CoS_2_@C result in varying adsorption energy values. For the single graphene layer model, the adsorption energies are −2.7 eV and −3.6 eV when lithium is located below (Figure 9b) and above (Figure 9c) graphene, respectively. Adding one more graphene layer in our simulated model increases the adsorption energy significantly, to −4.0 eV as shown in Figure 9d. Although our simulation has limitations in accurately representing multiple graphene layers, there is an evident trend that suggests an increase in adsorption energy with the addition of more graphene layers. This indicates that the presence of graphene‐like carbonaceous layers can further enhance the interaction between lithium and CoS*_x_*, potentially leading to improved lithiation efficiency.

## 3 Conclusion

In summary, this study has not only aimed to harness the respective advantages of cobalt and molybdenum sulfides but has also introduced innovative concepts and materials for the development of advanced anodic materials for LIBs. Through a straightforward two‐step hydrothermal process, we successfully synthesized the CoMoS composite. However, the true innovation lies in our approach of encapsulating the CoMoS nanocomposites within a carbonized polydopamine shell, denoted as CoMoS@NC, serving as both a buffering and conducting matrix. The main findings of our study reveal that the nitrogen‐doped carbon‐coated cobalt molybdenum sulfide composite, CoMoS@NC, surpasses its predecessor, CoMoS, in numerous aspects. CoMoS@NC exhibited an impressive de‐lithiation capacity of 709 mAh g⁻^1^, a substantial improvement compared to CoMoS (420 mAh g⁻^1^), even after enduring 100 lithiation/de‐lithiation cycles at a current density of 200 mA g⁻^1^. Moreover, the long‐term stability of CoMoS@NC was remarkable, with 94% of its capacity retained after 600 consecutive lithiation/de‐lithiation cycles. Furthermore, a comprehensive evaluation in a LIB full‐cell configuration, pairing CoMoS@NC with an NMC‐532 cathode, highlighted its potential as a next‐generation LIB anode. Additionally, our DFT calculations revealed that graphene‐like carbonaceous layers enhance the interaction between lithium and metal sulfide, providing improved lithiation efficiency. Future work should focus on optimizing synthesis, exploring broader applications, and delving into the underlying electrochemical mechanisms to advance energy storage materials.

## Statistical Analysis

The output performances such as lithiation–delithiation capacities, CVs, EIS, differential capacities, etc were all adopted directly (without any pre‐processing) from the measured original data using the electrochemical workstations mentioned in the electrochemical characterization section. All the data plotting was performed using Origin (software).

## Conflict of Interest

The authors declare no conflict of interest.

## Author Contributions

R.M.B.: Conceptualization, Methodology, Investigation, Visualization, Data curation, Writing‐original draft; N.L., K.C. and D.A.: Software, Formal analysis, Characterization, Validation; S.M.S.P., S.S.: Validation, Writing‐review and editing; S.J.K.: Investigation; Resources; Validation; Y.S.M.: Supervision, Writing‐review and editing, Resources, Funding acquisition, Validation.

## Supporting information

Supporting Information

## Data Availability

The data that support the findings of this study are available from the corresponding author upon reasonable request.
