# Supplementary material for: Synergistic Performance Boosts of Dopamine‐Derived Carbon Shell Over Bi‐metallic Sulfide: A Promising Advancement for High‐Performance Lithium‐Ion Battery Anodes
Source: Adv Sci (Weinh). 2024 Feb 11;11(15):2308160. doi: 10.1002/advs.202308160 (PMC11022702; doi:10.1002/advs.202308160)
Supplement: Supplementary file 1 — Supporting Information [file ADVS-11-2308160-s001.pdf]

ADVANCED SCIENCE
Open Access

Supporting Information

for *Adv. Sci.*, DOI 10.1002/advs.202308160

Synergistic Performance Boosts of Dopamine-Derived Carbon Shell Over Bi-metallic Sulfide: A Promising Advancement for High-Performance Lithium-Ion Battery Anodes

*Roshan Mangal Bhattarai, Nghia Le, Kisan Chhetri, Debendra Acharya, Sudhakaran Moopri Singer Pandiyarajan, Shirjana Saud, Sang Jae Kim and Young Sun Mok**

Synergistic Performance Boosts of Dopamine-Derived Carbon Shell over Bi-metallic Sulfide: A Promising Advancement for High-Performance Lithium-Ion Battery Anodes

Roshan Mangal Bhattarai[1], *Nghia Le*[2], *Kisan Chhetri*[3,4], *Debendra Acharya*[3], *Sudhakaran Moopri Singer Pandiyarajan*[5], *Shirjana Saud*[1], *Sang Jae Kim*[6], *Young Sun Mok*[1*]

[1]Department of Chemical Engineering, Jeju National University, 102 Jejudaehak-ro, Jeju 63243, Republic of Korea.

[2]Department of Chemistry, Mississippi State University PO Box 9573, Mississippi State, MS 39762 USA.

[3]Department of Nano Convergence Engineering, Jeonbuk National University, Jeonju-561756, Republic of Korea.

[4]Regional Leading Research Center (RLRC) for Nanocarbon-based Energy Materials and Application Technology, Jeonbuk National University, Republic of Korea.

[5]School of Materials Science & Engineering, Kookmin University, Seoul 02707, Republic of Korea.

[6]Nanomaterials and System Laboratory, Department of Mechatronics Engineering, Jeju National University, 102 Jejudaehak-ro, Jeju 63243, Republic of Korea.

*Corresponding author: smokie@jejunu.ac.kr ; youngsunmok@gmail.com

Tel.: +82-064-754-3682; Fax: +82-64-755-3670

1 Experimental

1.1 Materials and Method

All chemicals used in the process were of research-grade and were used as they arrived without further purification. Cobalt nitrate ($Co(NO_3)_2.6H_2O$) and sodium molybdate ($Na_2MoO_4.2H_2O$) were purchased from Junsei Chemical Co., Ltd., Japan. Thioacetamide (C_2H_5NS) was purchased from Wako pure chemical, Japan. Dopamine hydrochloride was purchased from Sigma Aldrich, Germany. Tris buffer was purchased from Alfa Aesar, China. Ethanol (C_2H_5OH) was purchased from Daejung Chemicals & Metals Co., Ltd., South Korea. Extra pure deionized (DI) water was used throughout the experiment. JAC ULTRASONIC-4020 series was used for the ultrasonication work.

1.2 Preparation of $CoMoO_4$

In a typical synthesis process, 1 mmol of $Co(NO_3)_2.6H_2O$ and 1 mmol of $Na_2MoO_4.2H_2O$ were added in 80 mL solution of solvents composed of water, ethanol and diethyl glycol in the volumetric ratio of 2:1:2. The solution was stirred in magnetic stirrer for 1 h then kept in 100 ml Teflon-lined steel autoclave and heated at 180 °C for 6 h. The final solution was obtained as a pink segregate in a clear solution. The obtained solution was washed several times with ethanol and DI water and kept in a vacuum oven at 80 °C for 8 h to dry. Dark violet (color code: #9400d3) powder was obtained after vacuum drying. This powder is believed to be the cobalt molybdenum hydroxide. The product obtained from vacuum drying was then kept in a high-temperature furnace at 400 °C at the rate of 1 °C per minute for 3 h for the final annealing process. Dark blue magenta (color code: #685580) powder was obtained after the final calcination process. The final product was named $CoMoO_4$.

1.3 Preparation of CoMoS

CoMoS composite was synthesized through the second step hydrothermal treatment of $CoMoO_4$ using thioacetamide as the sulfur source. In a typical process, $CoMoO_4$ powder (100 mg) and thioacetamide (300 mg) were mixed for 30 min in 40 ml DI water. The mixed solution was then put in an oven at 120 °C for 5 h in the hydrothermal vessel. The final product obtained in the form of a black suspension was centrifuged and washed several times with DI water and ethanol and dried in a vacuum oven at 80 °C overnight. The final product was ground again to make the fine powder for further use and named CoMoS-3. For ease of literature writing and understanding, the sample names CoMoS and CoMoS-3 are used interchangeably and hold the same meanings. As a control sample, the cobalt molybdenum sulfide was prepared using different $CoMoO_4$ and thioacetamide powder weight concentrations such as 1:1, 1:2, and 1:4 and the samples obtained were named CoMoS-1, CoMoS-2, and CoMoS-4 respectively. Furthermore, a solid-state synthesis method was also employed to synthesize the cobalt molybdenum sulfide, where, a 1:3 weight ratio of $CoMoO_4$ and thioacetamide was ground together and calcined at 500 degrees to get the cobalt molybdenum sulfide. Due to its morphological non-homogeneity (Figure S1), the solid-state synthesized cobalt molybdenum sulfide was not used for further processes. Furthermore, as a control sample, the CoMoS was also prepared with a one-step hydrothermal approach, where Co, Mo, S precursors (1 mmol $Co(NO_3)_2.6H_2O$, 1 mmol of $Na_2MoO_4.2H_2O$, and 2mmol of thioacetamide in 80 mL solution of solvents composed of water, ethanol and diethyl glycol in the volumetric ratio of 2:1:2) were mixed in a single Teflon lined hydrothermal vessel and heated at 180 °C for 6 h. The final product obtained after consecutive washing and drying is named CoMoS-H.

1.4 Preparation of CoMoS@NC

We implemented the self-polymerization ability of dopamine to form a very thin carbon film on CoMoS nanoparticles. In a typical process, 200 mg of CoMoS was dispersed on tris buffer solution (pH 8.5) and left to sonicate for 1 h. After the sonication, 100 mg dopamine hydrochloride was added to the solution and left to stir overnight in a magnetic stirrer. The dark black solution obtained after stirring was washed with DI water a few times and then with ethanol. The final black compound was kept in a vacuum oven at 80 ℃ for drying. The vacuum-dried black compound was kept in a furnace at 500 ℃ for 4 h at 3 ℃ /min under an argon atmosphere for calcination. The final black powder obtained was named CoMoS@NC. A similar process was followed to obtain the CoMoS@NC-H, which is nitrogen-doped carbon coated CoMoS-H.

2 Characterization

2.1 Physical characterization

The nanocomposites obtained were characterized using different approaches like X-ray diffraction (XRD) analysis by using the PANanalytical’s Empyrean XRD with Cu Kα (λ = 0.15405 nm) radiation in the scan range (2θ) of 10° to 80°. The structural morphologies of the samples were observed with a field emission scanning electron microscope (FE-SEM) (TESCAN, MIRA3), and chemical compositions were evaluated by energy-dispersive spectroscopy (EDS) measurements and element mapping (TESCAN, MIRA) measurements at 15 kV. High-resolution (HR) brightfield imaging and combined high-angle annular dark-field (HAADF) scanning were also performed with Thermo Scientific Talos F200X G2 (Jeju National university, South Korea). X-ray photoelectron spectroscopy (XPS) was conducted using a Theta Probe K-ALPHA+XPS

system (Thermo Fisher Scientific.) with monochromatic Al Kα at a wavelength of 1486.6 eV at 12 kV, KBSI (Korea Basic Science Institute, Busan Center).

2.2 Electrochemical Characterization

The working electrodes were prepared by using a slurry-coating procedure. The slurry was prepared by mixing 70 wt.% active material (CoMoS or CoMoS@NC), 15 wt.% carbon black, and 15 wt.% CMC binder using DI water as a solvent. The mixture was hand-ground with a pestle in a mortar until there were no granular particles remained. Then the slurry was casted onto a copper foil and dried at 110°C for 10 h under vacuum. Thus, prepared copper foil coated with active material was clamped with 12 mm dia. crimper for an electrode. Test cells (CR 2032) were assembled in an argon-filled glove box with a metallic lithium foil as a reference electrode, 1M $LiPF_6$ electrolyte dissolved in ethylene carbonate/dimethyl carbonate (EC: DMC=1:1 v/v), and a Celgard 2400 polypropylene (PP) microporous film as a separator. Galvanostatic charge/discharge tests were conducted on PESC05-0.1 PNE Power and Energy Solution cell cycler in the voltage range of 0.01–3.0 V at a controlled room temperature of 25°C.

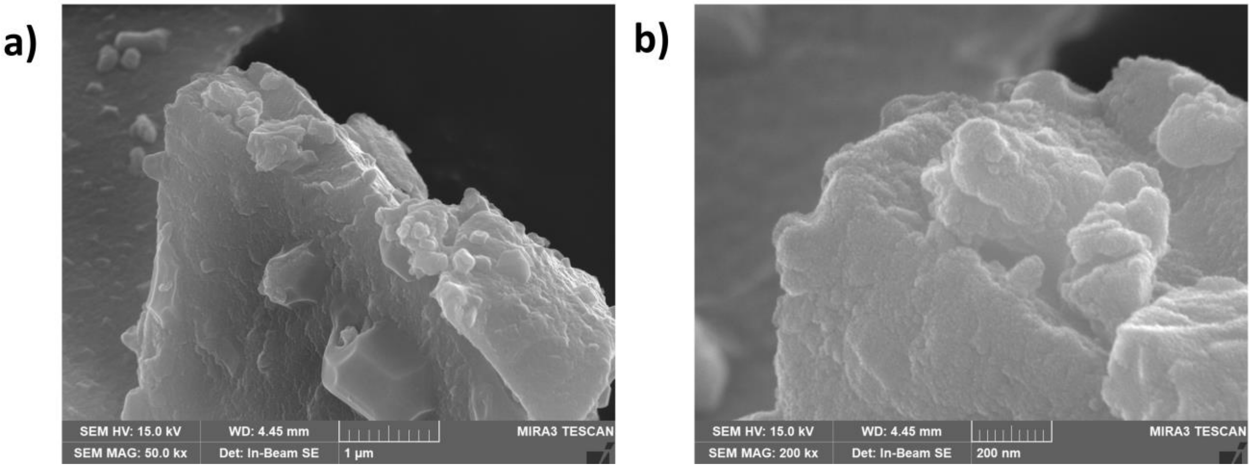

Fig. S1. FE-SEM images of high-temperature solid-state sulfurization under different magnifications

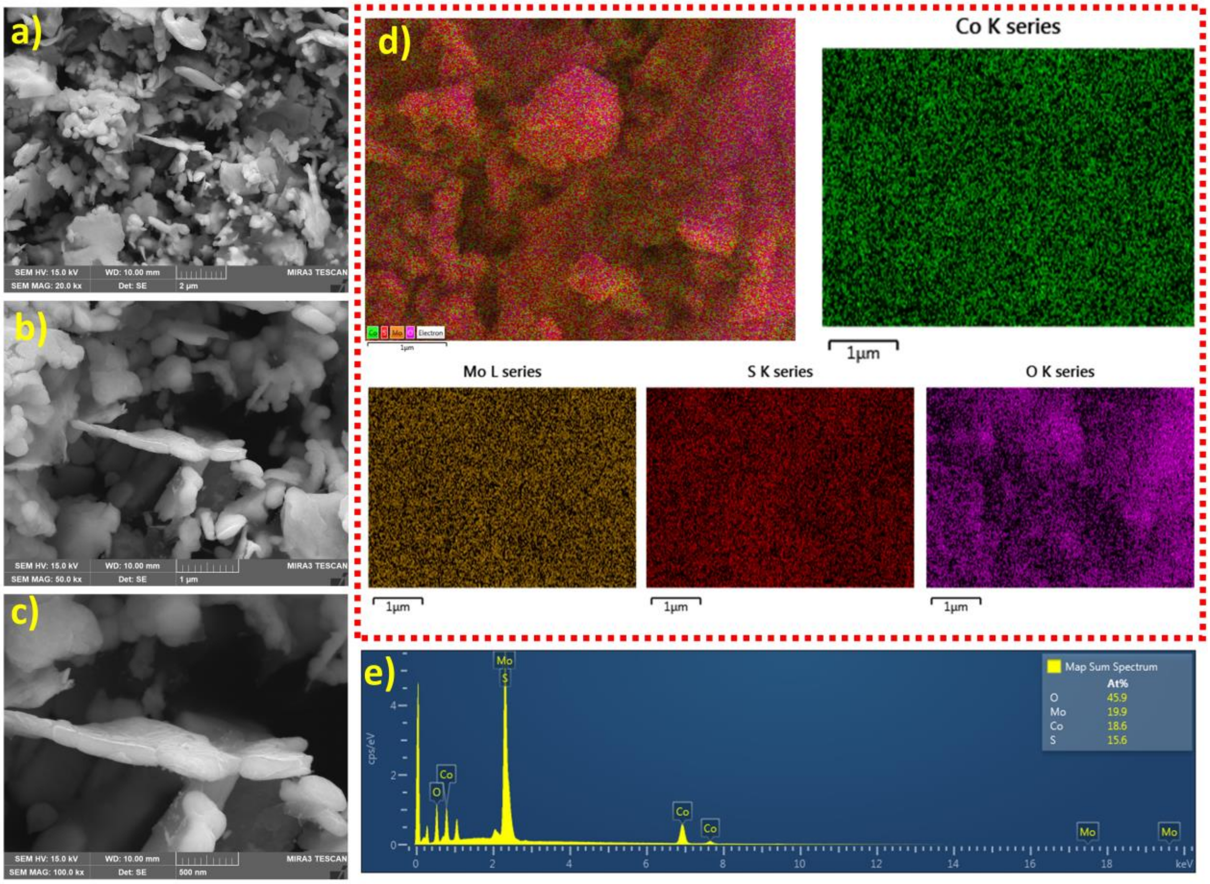

Figure S2 a-c) FE-SEM images, d)EDS elemental color mapping, and e) EDS spectrum of CoMoS-H

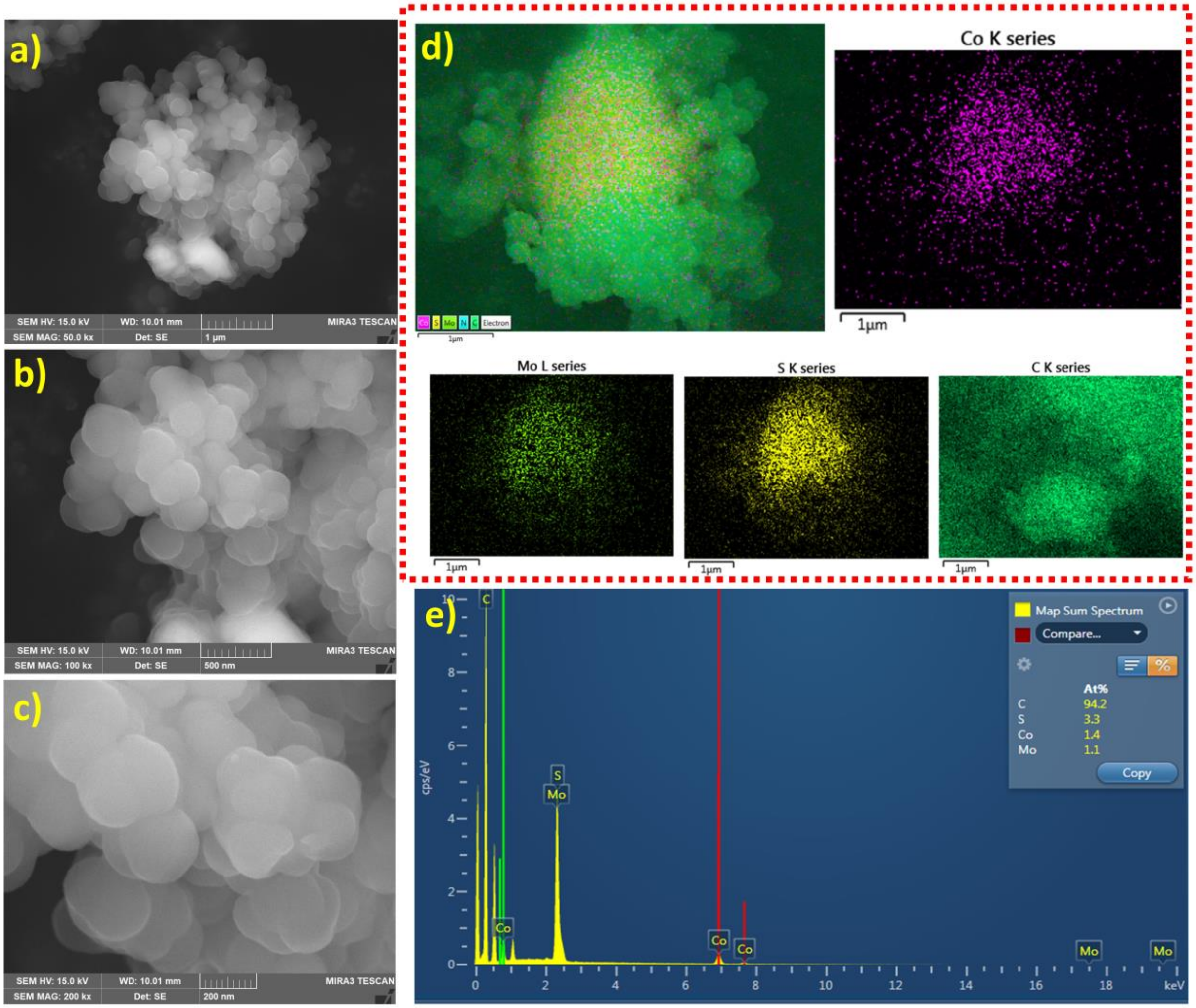

Figure S3 a-c) FE-SEM images, d)EDS elemental color mapping, and e) EDS spectrum of CoMoS@NC-H

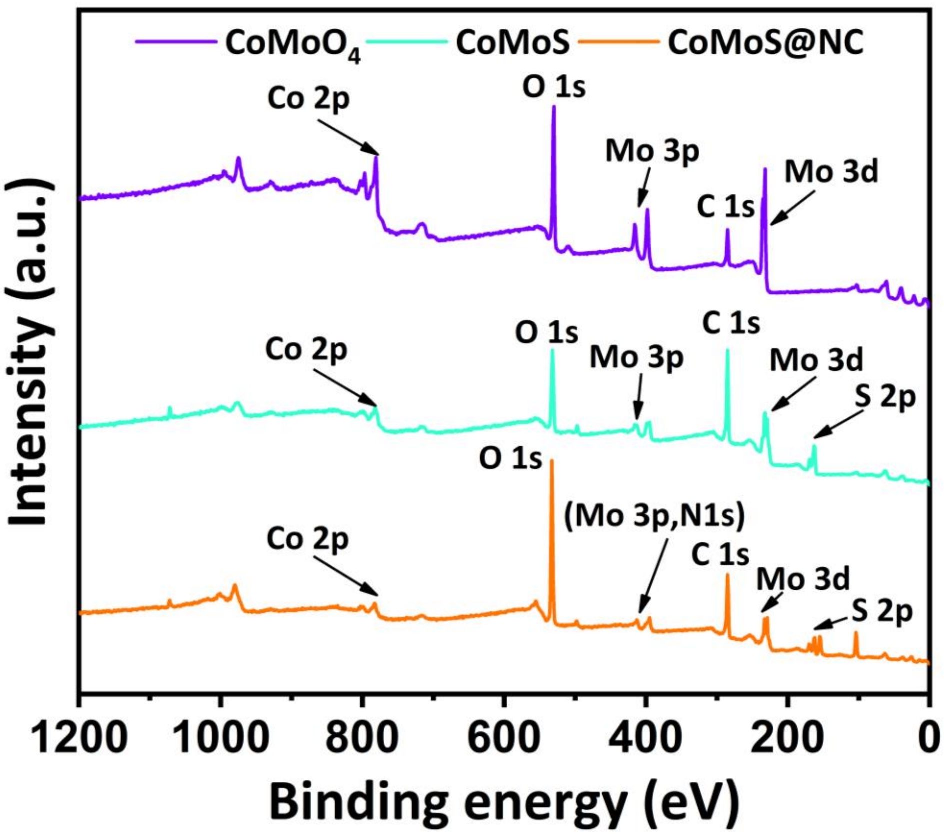

Fig. S4 XPS survey spectra of $CoMoO_4$, CoMoS, and CoMoS@NC

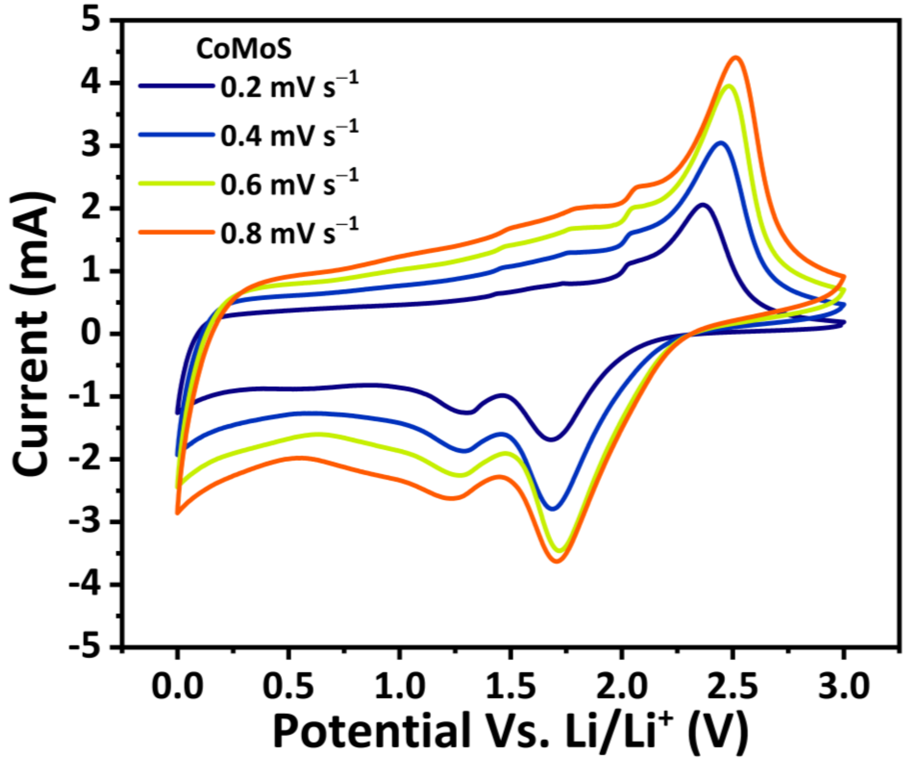

Fig. S5. CV curves of CoMoS cell over different scan rates

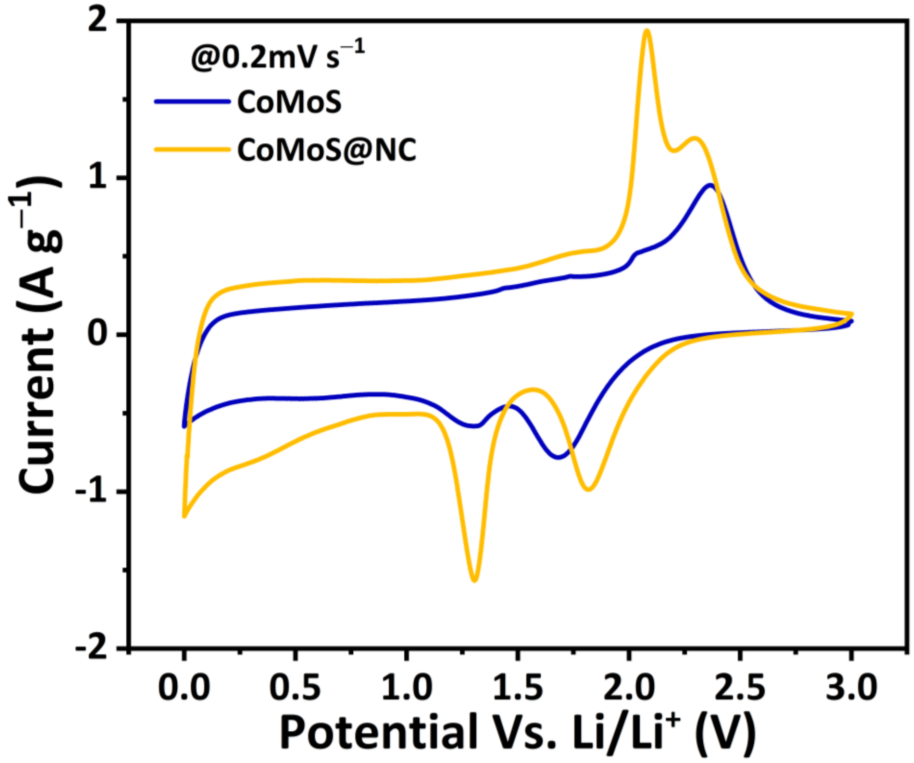

Fig. S6. Mass specific CV of CoMoS and CoMoS@NC at 0.2 mV s^{-1}

a)

Equation	y = a + b*x					
Plot	line1	line2	line3	line4	line5	line6
Intercept	2.2087 ± 0.04	2.03491 ± 0.	2.39383 ± 0.0	2.09764 ± 0.0	2.05517 ± 0.0	2.35313 ± 0.2
Slope	0.69335 ± 0.0	0.53852 ± 0.	0.56185 ± 0.0	0.58422 ± 0.0	0.52752 ± 0.0	0.56973 ± 0.0
Adj. R-Sq	0.99899	0.99955	0.99371	0.9953	0.9971	0.94914

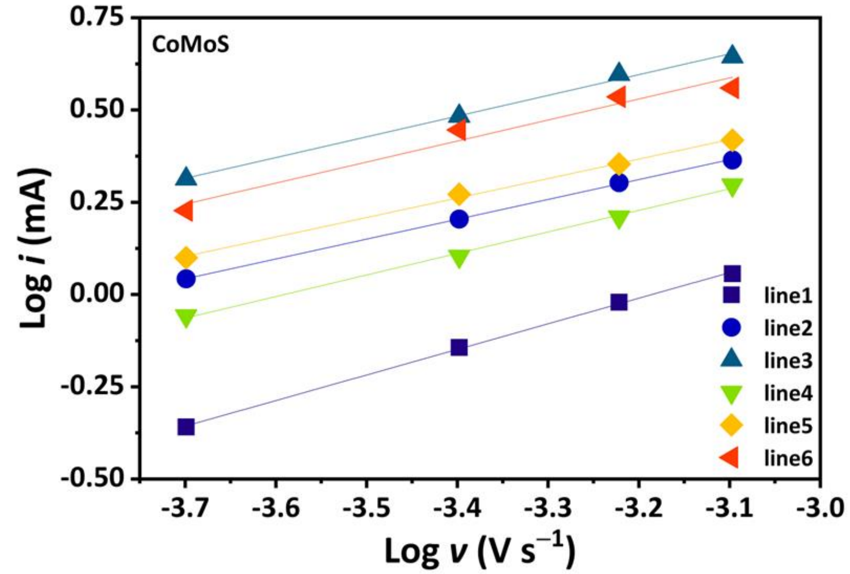

b)

Equation	y = a + b*x					
Plot	line1	line2	line3	line4	line5	line6
Intercept	2.55671 ± 0.08	2.05853 ± 0.08	3.16064 ± 0.02	2.75542 ± 0.02	2.27091 ± 0.04	2.91067 ± 0.04
Slope	0.83565 ± 0.02	0.50068 ± 0.02	0.84842 ± 0.00	0.82484 ± 0.00	0.58269 ± 0.01	0.80826 ± 0.01
Adj. R-Squ	0.99698	0.99281	0.99967	0.99975	0.99871	0.99907

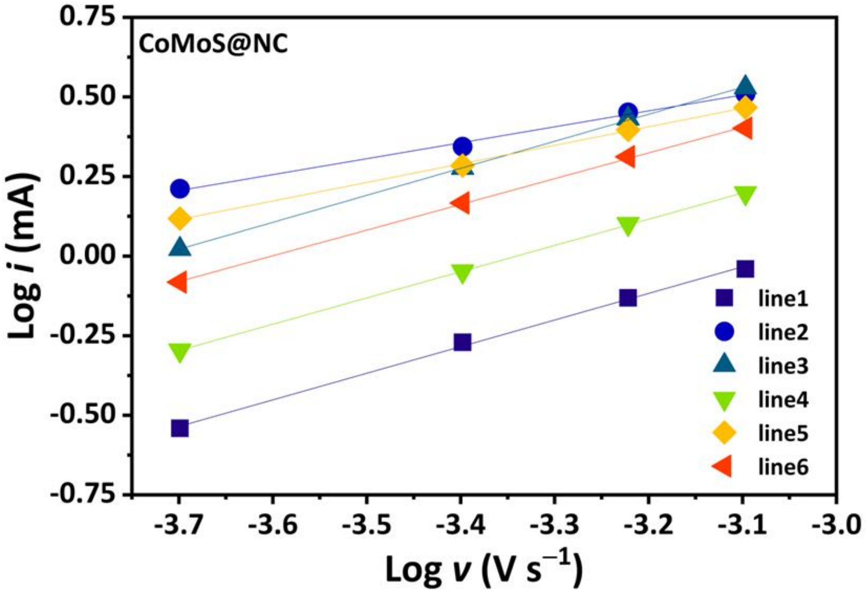

Fig. S7. b-value calculation for (a) CoMoS and (b) CoMoS@NC at different points represented by the line numbers

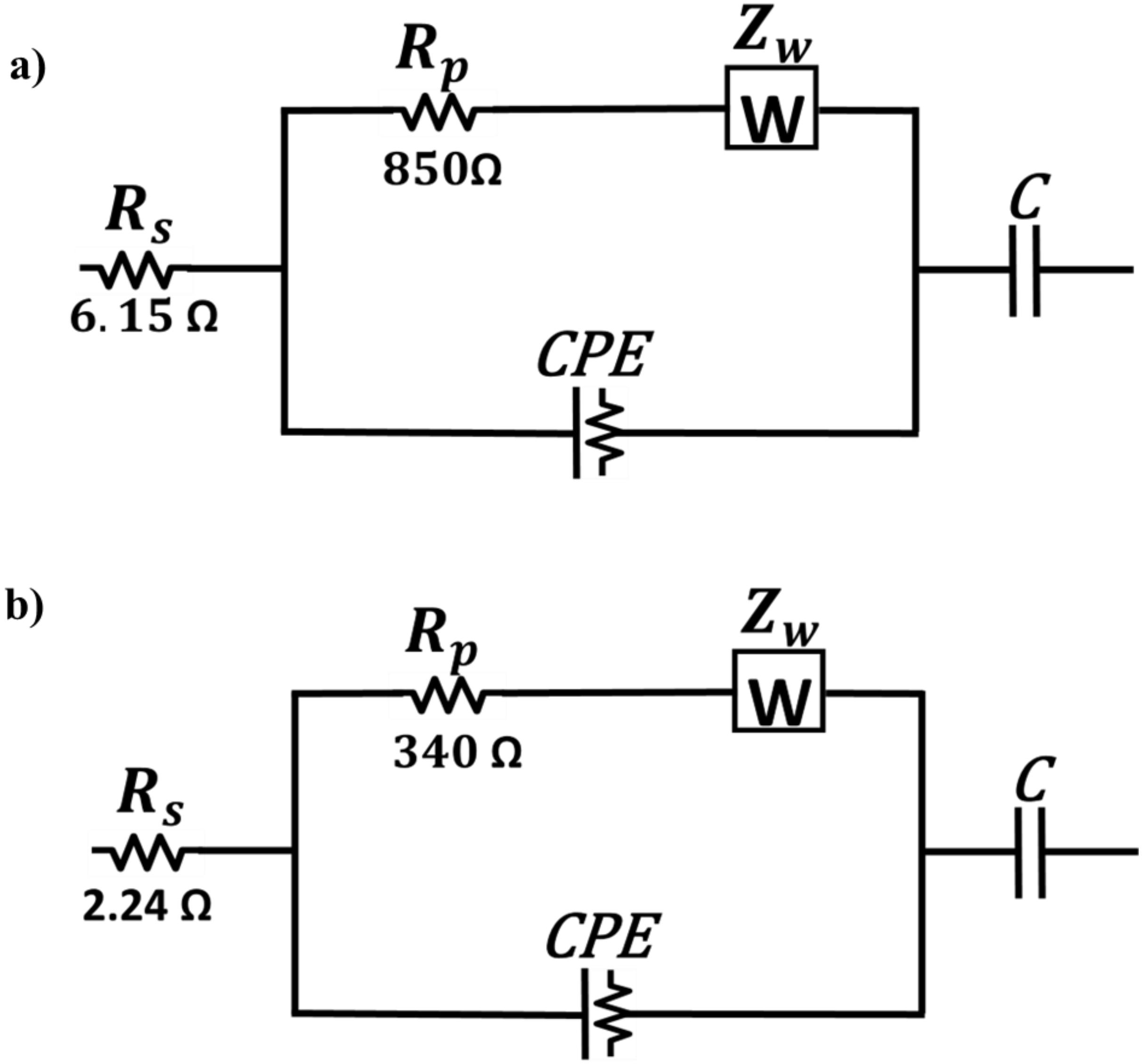

Fig. S8. EIS equivalent electrical circuit of a) CoMoS and b) CoMoS@NC with relevant equivalent electrical parameters

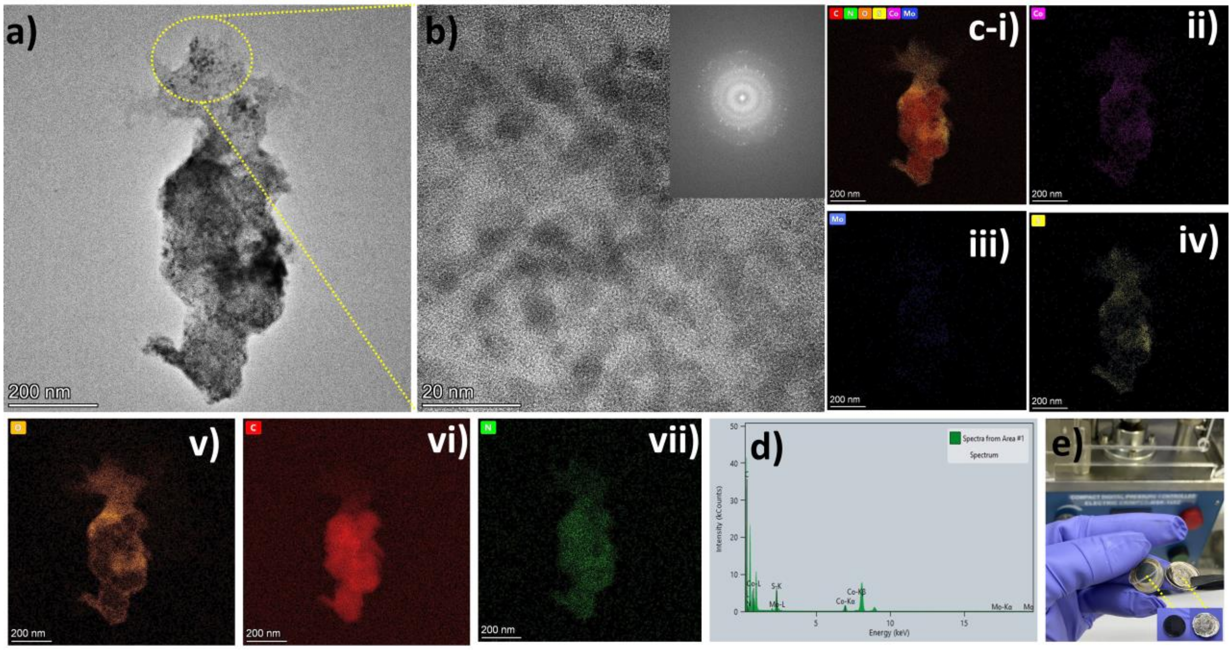

Figure S9 a)TEM and b)HR-TEM, c i-vii) EDS elemental color mapping, d) EDS elemental spectrum of CoMoS@NC after 600 cycles. e) electronic image of de-assembled CoMoS@NC coin cell after 600 cycles.

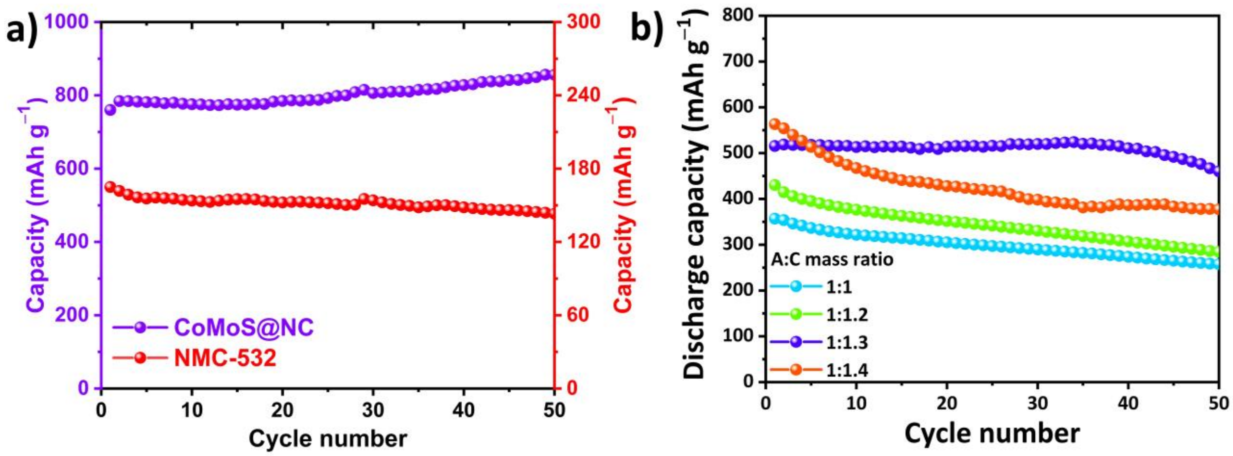

Fig. S10 (a) Discharge capacity of CoMoS@NC and NMC-532, and (b) Discharge capacity of a full cell over different anode to cathode AM mass ratios.
